# Plasma and CSF biomarkers of aging and cognitive decline in Caribbean vervets

**DOI:** 10.1002/alz.14038

**Published:** 2024-07-01

**Authors:** Curran Varma, Eva Luo, Gustaf Bostrom, Praveen Bathini, Daniela Berdnik, Tony Wyss‐Coray, Tingting Zhao, Xianjun Dong, Frank R. Ervin, Amy Beierschmitt, Roberta M. Palmour, Cynthia A. Lemere

**Affiliations:** ^1^ Department of Neurology Ann Romney Center for Neurologic Diseases Brigham and Women's Hospital Boston Massachusetts USA; ^2^ Department of Neurology Harvard Medical School Boston Massachusetts USA; ^3^ Department of Public Health and Caring Sciences Geriatrics, Uppsala University Uppsala Sweden; ^4^ Centre for Clinical Research Uppsala University Västmanland County Hospital Västerås Sweden; ^5^ Department of Neurology and Neurological Sciences Stanford University School of Medicine Stanford California USA; ^6^ Department of Medical Oncology Dana‐Farber Cancer Institute Boston Massachusetts USA; ^7^ Genomics and Bioinformatics Hub Brigham and Women's Hospital Boston Massachusetts USA; ^8^ Behavioral Sciences Foundation Saint Kitts, Eastern Caribbean Montreal Canada; ^9^ Faculty of Medicine and Health Sciences McGill University Montreal Canada; ^10^ Department of Biomedical Sciences Ross University School of Veterinary Medicine St Kitts UK

**Keywords:** aging, Alzheimer's disease, biomarkers, CSF, dementia, non‐human primates, omics, plasma, proteomics, vervets

## Abstract

**INTRODUCTION:**

Vervets are non‐human primates that share high genetic homology with humans and develop amyloid beta (Aβ) pathology with aging. We expand current knowledge by examining Aβ pathology, aging, cognition, and biomarker proteomics.

**METHODS:**

Amyloid immunoreactivity in the frontal cortex and temporal cortex/hippocampal regions from archived vervet brain samples ranging from young adulthood to old age was quantified. We also obtained cognitive scores, plasma samples, and cerebrospinal fluid (CSF) samples in additional animals. Plasma and CSF proteins were quantified with platforms utilizing human antibodies.

**RESULTS:**

We found age‐related increases in Aβ deposition in both brain regions. Bioinformatic analyses assessed associations between biomarkers and age, sex, cognition, and CSF Aβ levels, revealing changes in proteins related to immune‐related inflammation, metabolism, and cellular processes.

**DISCUSSION:**

Vervets are an effective model of aging and early‐stage Alzheimer's disease, and we provide translational biomarker data that both align with previous results in humans and provide a basis for future investigations.

**Highlights:**

- We found changes in immune and metabolic plasma biomarkers associated with age and cognition.
- Cerebrospinal fluid (CSF) biomarkers revealed changes in cell signaling indicative of adaptative processes.
- TNFRSF19 (TROY) and Artemin co‐localize with Alzheimer's disease pathology.
- Vervets are a relevant model for translational studies of early‐stage Alzheimer's disease.

## 1 BACKGROUND

Alzheimer's disease (AD) is the leading cause of dementia in older adults. Coupled with an aging population, this disease is on track to becoming increasingly prevalent with 152 million cases projected by 2050.^1^ Early‐onset familial autosomal dominant accounts for ≤5% of AD cases and is due to genetic mutations in the amyloid precursor protein (*APP*) or presenilin 1 or 2 (*PSEN1, PSEN2*) genes, all of which play a role in amyloid beta (Aβ) generation, whereas late‐onset AD (LOAD) accounts for the remaining 95% and is sporadic.^2^ Age remains the most significant risk factor for LOAD,^3^ but pathology begins decades before clinical symptoms are apparent.^4^ In the past, patients presenting AD‐like symptoms could not be definitively diagnosed with AD until after their death when an autopsy was performed and pathology could be observed. Currently, other methods, such as blood tests, analysis of known AD markers in cerebrospinal fluid (CSF) (Aβ and tau), positron emission tomography (PET), computed tomography (CT), or magnetic resonance imaging (MRI) scans, and cognitive tests, are performed to rule out other possibilities and point to the diagnosis.^5^, ^6^ As the number of AD cases increases dramatically, accurate, predictive, and accessible measures, as well as novel therapeutic targets, will be essential for faster screening and longitudinal evaluations. CSF analysis can provide particularly useful metrics due to the benefit of being connected to the brain parenchyma (and central nervous system), whereas blood tests are an example of a less invasive method that would allow for more patients to be diagnosed and consistently monitored. Despite the immense impact that AD's prevalence will have on health care systems and families around the world, there exists no treatment that can completely cure or prevent the disease.^7^ Given that an early diagnosis can allow patients to begin medications that can slow AD progression^8^ and provide families with more time to plan for future care, the few treatment options combined with an extended pre‐clinical period call for the discovery of reliable, predictive biomarkers. Many AD studies have used small animal models, such as mice, as they are relatively more accessible, have short lifespans, and allow for transgenic and knock‐in models expressing human AD genes. Although valuable, given that these models are genetically modified to exhibit AD pathology, they represent familial AD, which only accounts for <5% of cases in humans.^9^ Non‐human primates (NHPs) are excellent models for many human diseases, as their close phylogenetic relationship to humans means they model biological and behavioral functions similarly, and AD is no exception. Caribbean populations of African green vervet monkeys (*Chlorocebus aethiops*) are genetically 96% homologous to humans^10^ and were used in this study as a model for aging and the natural development of AD pathology. Vervets are genetically “clean,” as they do not carry any pathogenic viruses from Africa and are non‐endangered.^11^ They can live up to 20 years old (YO) in the wild and around 30 YO in captivity.^12^ For reference, a 23 YO vervet is roughly equivalent to an 80 YO human. Apolipoprotein ε4 (apoE4) is a cholesterol transporter and the greatest genetic risk factor for AD after aging.^13^ Although a single allele increases one's risk by up to 3‐fold, two alleles can increase risk as much as 15‐fold.^13^ NHPs are 100% homologous for APP, and vervets are homozygous for apoE4; consequentially, they develop AD pathology, such as Aβ plaques, in a manner similar to humans.^14^, ^15^, ^16^, ^17^ Previous studies have shown vervets to develop Aβ plaques histologically similar to those seen in humans and have declining levels of CSF Aβ42 and Aβ40 with age and plaque density, as also seen in humans.^17^ Furthermore, vervet gait speeds decline with age and CSF Aβ42 levels and neuroimaging via MRI and ^18^F‐FDG (fluorodeoxyglucose) PET has revealed older animals with greater plaque burden to have reduced volumes and cerebral metabolic rates of glucose in several brain regions.^17^ Using immunohistochemistry (IHC) on a large sample of archived brain tissue from male and female vervets with ages spanning their lifespan, we provide robust evidence of age‐dependent increases in AD pathology within our cohort including many vervets near the end of their lifespan, when there are higher incidences of cognitive impairment, compared to other studies. We then wondered if there were proteins in the blood and CSF of vervets that were significantly associated with the processes of aging and cognitive decline. To answer this question, we used proteomics in cross‐sectional plasma samples and longitudinal CSF samples to identify biomarkers that significantly change with age, sex, cognition, and CSF Aβ levels (with plasma biomarkers). Few omics studies have been done in vervets and even fewer in the context of aging and AD.^18^, ^19^, ^20^, ^21^, ^22^ In addition, many vervet studies have either solely or mostly examined female subjects. To our knowledge, this is the first study to use large‐scale proteomics in the plasma and CSF of male and female vervets and analyze the results in relation to key variables. We identified numerous biomarkers in both plasma and CSF that demonstrated significant associations with aging and cognitive performance, many of which have previously shown similar results in humans and others that provide a basis for future studies to explore new biomarkers not previously shown to have such associations. Overall, we provide substantial evidence that vervets are a translational model of aging, early‐stage AD, and Aβ pathology, and highlight changes in plasma and CSF biomarkers underlying aging and cognitive decline.

## 2 METHODS

### 2.1 Primate subjects

All vervets (*Chlorocebus aethiops*) included in the study came from St. Kitts, Eastern Caribbean. Those born in the Behavioural Sciences Foundation (BSF) colony had known ages, whereas the ages of feral vervets brought into the colony were estimated using physical characteristics and sexual maturity and had a margin of error of 2 years. The BSF laboratories are accredited by the Canadian Council for Animal Care and hold an OLAW (National Institutes of Health [NIH]) registration as well. All animals used in this study were housed in outdoor enriched social groups in the BSF laboratories. This setting resembles their natural environment and provides regular foraging opportunities. The animals were fed Harlan Teklad high protein primate chow (5% body weight/day) supplemented with local produce and fresh fruit. Water was available ad libitum. Mostly distinct cohorts of vervets were used for the pathology, CSF, and plasma biomarker studies. Further information regarding vervet age, sex, and cognitive scores can be found in Table S1.

#### 2.1.1 Vervet tissue preparation and processing

No vervets were euthanized to conduct pathology studies. Upon receiving formalin‐fixed tissues from the BSF archive, hemibrains were dissected rostral to caudal into nine distinct regions: prefrontal cortex, frontal cortex (FC), parietal lobe, temporal lobe, temporal/hippocampal (TC/HC), cerebellum, occipital lobe, basal ganglia, and thalamus. However, only the frontal cortex and TC/HC regions were examined for this study. Paraffin sectioning was performed for immunohistochemical analysis. This included cutting 25, serial 10‐micron sections from each paraffin block and mounting them on glass slides. Cryosectioning was also performed after freezing adjacent blocks of tissue in optimal cutting temperature embedding compound.

**RESEARCH IN CONTEXT**

1. **Systematic review**: The authors reviewed literature using traditional sources to provide background on the biomarker hits. Many of the significant biomarkers have previously been implicated in aging and/or cognition.
2. **Interpretation**: Our findings show vervets to be a useful model of aging and early‐onset Alzheimer's disease (AD). The results support previous findings and provide a basis for the investigation of additional biomarkers.
3. **Future directions**: The significant pathways and biomarkers, and their linker genes, should be studied further to better understand the mechanisms of aging and cognitive impairment. In addition, such studies will benefit from the involvement of vervets given their translation to humans.

### 2.2 Human tissue samples

Human brain samples were collected postmortem at the time of autopsy, after obtaining prior consent from the next of kin for research participation and in accordance with protocols approved by the Partners Human Research Committee at Brigham & Women's Hospital (Boston, MA). Blocks of parietal cortex and TC/HC were fixed for 2 h in 10% neutral buffered formalin, rinsed in Tris buffered saline (TBS), and processed for paraffin embedding. Ten‐micron thick parietal and TC/HC sections from humans were used for immunofluorescent studies. Parietal sections came from three aged, non‐demented controls (74 M, 80 M, and 87 F YO) and three AD patients (78 M, 81 F, and 91 F YO). TC/HC sections came from the same AD patients and a different set of three non‐demented controls (60 F, 64 M, and 70 M YO) due to a lack of available sections. Further details, such as medical histories, can be found in Table S2.

### 2.3 Antibodies and histological stains

Brain tissue samples were stained with antibodies (Abs) used to visualize neuropathology. Abs used include Mabs MBC 40 (1:1000, gift from Haruyasu Yamaguchi, Japan) and 2G3 (1:1000, ELAN Pharm.) for Aβ1‐40, Mabs MBC 42 (1:1000, gift from Haruyasu Yamaguchi, Japan) and 21F12 (1:1000, gift from Dennis J. Selkoe, Brigham and Women's Hospital, Harvard Medical School, Boston, MA) for Aβ1‐42, and Pab R1282 (1:1000, gift from Dennis J. Selkoe, Brigham and Women's Hospital, Harvard Medical School, Boston, MA) for pan‐specific Aβ. For immunofluorescence studies, primary Abs used include MAP2 (1:100, Invitrogen, MA5‐12826; neuronal microtubule associated protein 2), glial fibrillary acidic protein (GFAP) (1:250, Millipore Sigma, G3893; astrocytes), AT8 (1:100, Invitrogen, MN1020; phosphorylated tau Ser202/Ser205), conjugated anti‐β‐amyloid (1:200, BioLegend, 856508), FGF18 (1:200, Invitrogen, PA5‐106562; fibroblast growth factor‐18), TROY (1:200, Invitrogen, PA5‐116076 TNFRSF19/TROY), and Artemin (1:50, Bioss, BS‐0055R) with Alexa Fluor conjugated secondary Abs (1:375, Invitrogen, A21430; 1:375, Invitrogen, A32728). Stained sections were cover‐slipped on microscope slides using ProLong Gold antifade reagent with DAPI (Invitrogen, P36931).

#### 2.3.1 Immunohistochemistry (IHC)

IHC was performed as described previously.^12^ Briefly, paraffin sections were deparaffinized and rehydrated by washing with Histoclear (National Diagnostics, Atlanta, GA) and graded ethanol (100%, 95%, 75%, 50%). Thirty percent H_2_O_2_ and methanol were used to quench endogenous peroxidases in the sections. Sections were then pretreated with specific methods depending on the primary antibody. Formic acid pretreatment was required for all Abs. Sections were then blocked in 10% normal serum in TBS for 20 min at room temperature (goat serum for Pab and horse serum for Mab). After the primary Abs were added they were incubated at 4°C overnight. After washing in TBS, a biotinylated secondary specific to the primary 1:200 (Vector Laboratories, Burlingame, CA) was added for 30 min. Samples were then incubated with an avidin peroxidase ELITE kit (Vector Laboratories) for another 30 min. Following another wash in 50 mmol/L Tris, the sections were visualized using activated 3, 3′diaminobenzidine (DAB; Sigma Chemical, St. Louis, MO). Sections were then counterstained using hematoxylin, differentiated in acid alcohol, cleared in Histoclear (National Diagnostics), and coverslipped with Permount (Fisher Scientific, Pittsburgh, PA).

#### 2.3.2 Quantification and image analysis

Sections stained for general Aβ, Aβ1‐40, and Aβ1‐42 in both the FC and TC/HC regions were all imaged using a Zeiss Axiovert Imager.A1 with an AxioCam MRc5 camera. For each section, the two regions with the highest density of plaques were imaged under a 10× objective. On average, two sections were imaged for each brain region. ImageJ (version 2.1.0) was used to quantify the percent‐area of tissue covered by plaques or diffuse amyloid in each image, and the average percent‐area was calculated for each vervet. Spinning disk confocal microscopy was used for immunofluorescent imaging. Images were taken using a Nikon Eclipse Ti under a 40× objective and ImageJ was used to quantify immunoreactivity. On average, two sections for each vervet were imaged for FGF18 and TROY staining, and one vervet and one human section (parietal and TC/HC each) were imaged for Artemin staining.

### 2.4 CSF and plasma collection

CSF was collected via cisternal puncture from fasted animals in St. Kitts anesthetized with ketamine (10 mg/kg) according to standard operating procedures. Aliquots of CSF were dispensed into cryovials (Corning) and frozen immediately on dry ice. Whole blood (5 mL) was then collected from the same animals by femoral venipuncture and immediately transferred into vacutainer tubes containing ethylenediaminetetraacetic acid (EDTA) as anticoagulant and rotated for mixing. Plasma was obtained by centrifugation, aliquoted into cryotubes, and frozen at −60°C until shipping to the analytic laboratory.

#### 2.4.1 Plasma proteomic quantification

The plasma of 60 vervets (29 M, 31 F) ranging from 7 to 34 YO was cross‐sectionally analyzed. Metabolic and inflammatory proteins were selected for analysis by Rules‐Based Medicine (Austin, TX) using their Human Multi‐Analyte Profile v1.6 panels of Luminex‐based multiplex immunoassays, which included 114 analytes. Raw data can be found in Table S3. Measurements of these markers were then examined in relation to age, sex, and cognition, as well as CSF Aβ levels.

#### 2.4.2 CSF proteomic quantification

CSF samples were collected from 32 vervets (25 F, 7 M) ranging in age from 11 to 28 YO. Initially, 614 biomarkers were analyzed longitudinally in samples collected at three time points over 4 years (4 animals had a fourth collection making the sample total 100) using the Communicome platform, which included markers of cell signaling and neuroinflammation in humans. Relative protein abundance was measured with enzyme‐linked immunosorbent assay (ELISA)–grade antibodies, obtained from commercial sources, against human signaling proteins, which were printed in five replicates onto SuperEpoxy2 glass slides (Arrayit) with a robotic microarrayer (NanoPrint LM210, Arrayit) fitted with 16 946MP4B pins. Arrays were vacuum‐sealed and stored at 4°C until use. Samples were biotinylated on primary amino groups (EZ‐link NHS‐Biotin, ThermoScientific) overnight at 4°C and unbound biotin was removed via phosphate‐buffered saline (PBS) dialysis. Arrays were then blocked with 3% (w/v) casein solution before incubation with the individual biotinylated samples overnight at 4°C. After the washes, arrays were then incubated with Alexa Fluor 555‐conjugated streptavidin secondary antibodies (Life Technologies) and a GenePix 4400A scanner with GenePix Pro7 software (Molecular Devices) was used to detect fluorescent signals. After initial data clean‐up, which removed extreme outliers and values below minimum cutoffs, the data were analyzed cross‐sectionally. For example, if for a given biomarker, one vervet had a single timepoint remaining and another had two, all three timepoints were treated as individual samples. Biomarkers detectable in fewer than 40 (of an initial 100) samples were excluded, leaving 536 analytes. Raw data can be found in Table S4. Correlation between these biomarkers and age, sex, and object retrieval testing (ORT) score was then examined.

### 2.5 Object retrieval testing (ORT)

Cognitive performance was evaluated using a modification of the object retrieval task of Diamond.^23^, ^24^ At the Behavioral Science Foundation (St. Kitts), this test utilized a transparent acrylic glass box with an open side (7.6 cm × 7.6 cm × 3.8 cm) fixed on a tray (53.3 cm × 20.3 cm) that was positioned in front of the subject animal in the home cage. No anesthesia, restraint, or food or water restriction was used during testing. The object of the test was for the animal to retrieve a treat positioned within the test box without hitting the transparent sides of the box. The treat could be positioned within the box at the front, middle, or back of the box and with the open side facing the animal or to either the right or left of the animal. As the test proceeded through the predefined five modules, the task was increasingly difficult. For each test period, there were three days of ungraded orientation, followed by up to 12 days of graded performance. Performance on each of the five modules was graded with a score of 2 associated with perfect performance, 1 with obtaining the treat, but hitting the side of the box, and 0 with failing to obtain the treat. The test was repeated every day for a maximum of 12 days after the orientation period. Animals that performed perfectly for 3 days running were considered to have “passed out” of the test and were assigned a score of 1–10 based on how long it took them to complete the challenge. Thus, an animal that performed perfectly on the first 3 days of graded participation would receive an ORT score of 1, one that performed perfectly on days 2–4 of graded participation would score 2, and so on. This is a score of the latency at which criterion (perfect score three times running) was achieved. It incorporates motor performance but does not account for partial success on individual modules of the task. To capture this information, we have added a metric defined as total success percentage (TSP), which was obtained by summing the animal's success on individual modules (as defined above) as related to the total number of modules attempted. In addition, the inter‐test interval was, on average, 2 years.

Although factor analysis of the first tests of this procedure suggests that the protocol is a good measure of attention, visual perception, and general intelligence, there are some important limitations to note. The first is that in some animals, there is definitely a learning curve, such that many animals take three or four repetitions (at 3‐ to 6‐month intervals) to perform at their maximum ability. Alternately, there are some (even‐aged animals) that immediately perform perfectly and some that never perform well. Cognitive impairment is not the only reason for poor performance: a few animals, particularly ones not born in our facility, are too neophobic to accept novel treats as safe. For multiple reasons, we do not use regular foodstuffs such as bananas or other fruits as the treat in this task. Animals whose cognitive performance declines after initial good performance are those that we can say with confidence are becoming cognitively impaired with age, but unfortunately the samples used in this study do not always mirror that developmental time.

### 2.6 Statistical analysis

Linear regression and *t*‐test analyses for pathology studies were done using GraphPad Prism version 9.3.1. Custom R scripts were created for bioinformatic analyses. Linear regressions were done to find biomarkers that linearly changed with age or ORT score. One‐way analyses of variance (ANOVAs) and *t*‐tests were done to compare biomarker levels between age and ORT score groups. *p*‐values were FDR and Bonferroni corrected for multiple testing. R code for bioinformatic analyses can be found at: https://github.com/cvcv23/Vervet‐Proteomics.

### 2.7 Pathway analysis

Pathways analyses were performed using Gene Ontology (GO) and Reactome using the clusterProfiler^25^, ^26^ and ReactomePA^27^ R packages, respectively. Inputs for GO analysis were ENSEMBL gene IDs that were converted from official Gene Cards symbols using g:Profiler. In addition, GO ontology was set to “ALL” to include molecular functions, cellular components, and biological processes. To reduce the redundancy of GO results, the “simplify” function from clusterProfiler was used. ReactomePA requires ENTREZ IDs, so the “bitr” function from the clusterProfiler package was used for conversion. For both analyses, *p*‐values were adjusted using FDR and the significance cutoffs were *p* < 0.05 and FDR < 0.1.

### 2.8 Clustering trajectories & LOESS

To standardize protein levels with age, z‐scores were calculated. Plasma proteins with fewer than 40 samples were excluded to ensure precise grouping. Clustering of proteins was then done using pairwise differences in Euclidian distance; unsupervised hierarchical clustering was performed using the Ward.D2 method. Locally estimated scatterplot smoothing (LOESS) regression was fitted for both CSF and included plasma proteins with the average of protein levels represented by the thickest line. Pathway analysis on each cluster was done using Reactome and GO, as described above.

### 2.9 Protein–protein interaction (PPI) networks

PPI networks were created using the ReactomeFIViz app in Cytoscape. Inputs were official gene symbols from Gene Cards (genecards.org). Some plasma biomarkers, primarily immunoglobulins and steroid hormones, lack gene symbols and were unable to be included in the network. Linker genes were included in the network but excluded from pathway analyses to avoid biasing results. Clusters were created using a spectral partition‐based network clustering algorithm as described by Newman (2006).^28^ ReactomeFI queries CellMap, Reactome, KEGG, NCI PID, Panther, and BioCarta for pathway enrichment. GO biological processes were also explored. Both were done for all clusters and the whole network.

## 3 RESULTS

### 3.1 Age‐related Aβ deposition in the FC and TC/HC of vervet brains

**FIGURE 1:**
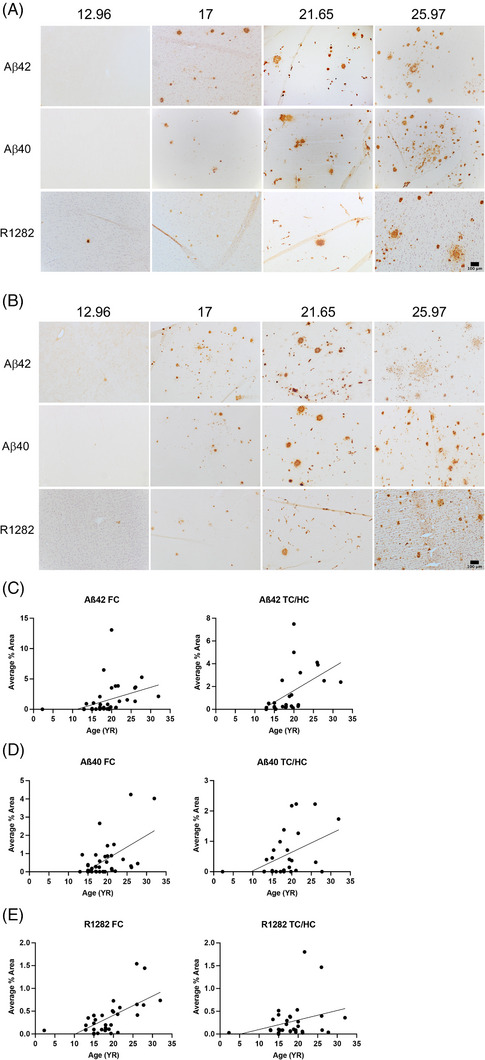
(A, B) Increases in Aβ pathology in the FC (A) and TC/HC (B) regions with age, ranging from 12.96 to 25.97 YO. Entire TC/HC sections were quantified by computerized image analysis; the images shown are from the TC. The same vervet is shown for each stain down each column, and the same vervets are shown for both brain regions. (C–E) Linear regressions of the average percent‐area of plaque from pathology analyses for Aβ42 (C), Aβ40 (D), and R1282 (E), for general Aβ. Scale bars: 100 μm.

C‐terminal Abs specific to Aβ ending at residues 42 and 40, as well as R1282, a polyclonal Ab that binds to multiple Aβ epitopes, were used to examine AD pathology development in the FC and TC/HC with age. The FC of 41 vervets (30 F, 11 M) ranging from 2.29 to 32 YO were stained with either MBC42 or 21F12 for Aβ42 immunoreactivity (IR); the same was done for the TC/HC of 25 vervets (18 F, 7 M) ranging from 12.92 to 32 YO. Significant increases in Aβ42 deposition were found in both the FC (*p* = 0.013) and TC/HC (*p* = 0.012) regions (Figure 1A,B,C). Abs MBC40 and 2G3 were used for Aβ40 IHC in the FC of 39 brains (29 F, 10 M), ranging from 12.96 to 32 YO, and the TC/HC of 29 brains (22 F, 7 M), ranging from 2.29 to 27.73 YO. Increases in Aβ40 deposition with age were again significant in both the FC (*p* = 0.0004) and TC/HC (*p* = 0.014) (Figure 1A,B,D). The FC of 33 brains (24 F, 9 M), ranging from 2.29 to 32 YO, and the TC/HC of 31 vervets (23 F, 8 M) with the same age range were used for R1282 IHC. Age‐related increases in Aβ were highly significant in the FC (*p* < 0.0001) but were found to be non‐significant (*p* = 0.116) in the TC/HC (Figure 1A,B,E).

Overall, Aβ plaques were more consistently found in vervets aged around 15 YO and older and vascular amyloid deposition was most frequently found in older vervets, beginning at 17 YO, with both Aβ42 and Aβ40 Abs indicating cerebral amyloid angiopathy (CAA).

### 3.2 CSF biomarkers

#### 3.2.1 CSF biomarkers of aging

**FIGURE 2:**
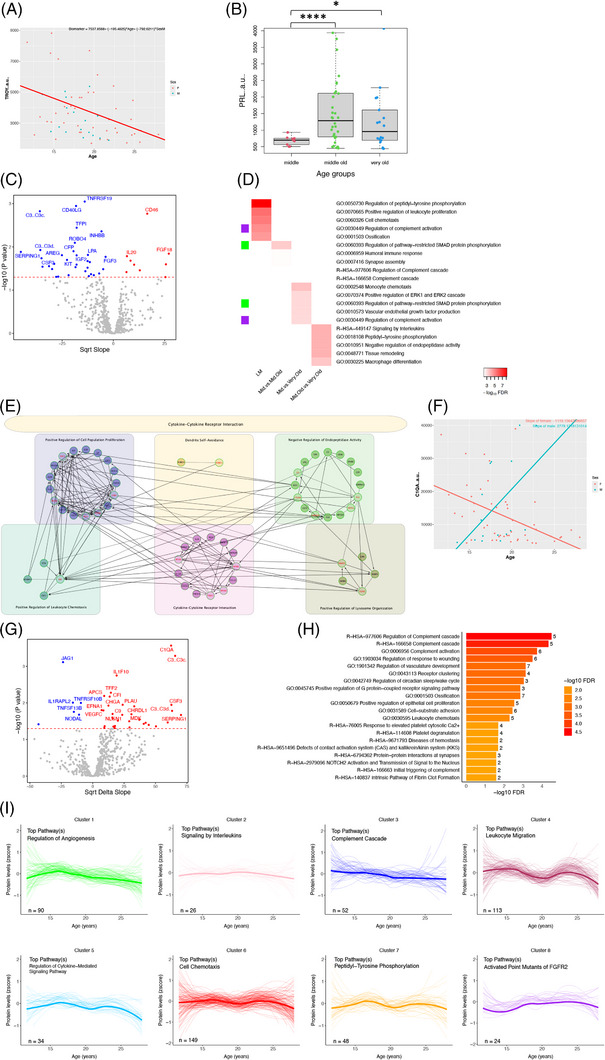
(A) Linear regression of the top hit with aging, adjusted for sex. (B) Box plot of PRL, which had the highest significance out of all age group comparisons among all markers. Asterisks indicate *p*‐value significance level (*p < 0.05, ****p < 0.0001). (C) Volcano plot of proteins with aging showing square root transformed slope and –log_10_ *p*‐value. Dotted red line indicates significance threshold. Red dots represent proteins that increased with age; blue dots decreased. (D) Heatmap of the top five pathways based on hits from each analysis; color indicates FDR significance level. Matching sidebar colors indicate overlapping pathways. (E) PPI network based on hits from linear model of general aging with linker genes (red labels) included. Colored boxes indicate identified clusters and are labeled with the top biological process/pathway; header indicates the same but for the whole network. (F) Top hit from sex differences with aging analysis. (G) Volcano plot of proteins showing sex differences with aging. Red dots indicate a higher slope in males; blue dots indicate higher in females. (H) Bar plot of top 20 pathways implicated in sex‐specific aging and the number of hits included in each pathway. (I) LOESS trajectories of z‐scores for eight clusters of proteins. Clusters were formed based on similarity of trajectories. Number of proteins (n) and most enriched pathway, based on GO and Reactome, are indicated. Arbitrary units (a.u.).

Linear regressions and *t*‐tests between age groups were done to find markers that changed significantly with age. Forty‐two markers demonstrated significant (*p* < 0.05) linear aging effects (Figure 2A,C). No marker passed multiple comparison corrections, although this is expected given our sample size and the large number of proteins included. Our analyses did yield numerous highly significant *p*‐values, however, and the top hit was TROY (TNFRSF19; *p* = 0.0009, FDR Q = 0.227), which declined with age (Figure 2A). Age groups of young (0‐10), middle (> 10‐15), middle‐old (>15‐20), and very‐old (>20) vervets were created to assess biomarker changes cross‐sectionally between different age groups. Following an ANOVA, *t*‐tests revealed TROY levels to be significantly decreased in the very‐old compared to the middle‐old age group (*p* = 0.026, FDR Q = 0.643) and nearly significantly lower when compared to the middle‐aged group (*p* = 0.057, FDR Q = 0.904). Previous work has not explored TROY's relationship with age; however, it is a tumor necrosis factor receptor superfamily member and has been implicated in cancer, vascular development in the brain, and blood–brain barrier functioning.^29^, ^30^ When looking at differences in the markers between age groups, there was a highly significant increase in prolactin (PRL) levels in the middle‐old compared to the middle‐aged group (*p* = 3.72E‐5, FDR Q = 0.02, Bonferroni Q = 0.02), as well as a relatively less, but still significant increase (*p* = 0.016, FDR Q = 0.79) in the very‐old group compared to the middle‐aged group (Figure 2B). PRL, best known for stimulating lactation, has additional roles in the central nervous system (CNS), which include effects on anxiety and depression as well as neuroprotection against inflammation.^31^, ^32^ Pathway analysis was performed for the hits of the linear model and age group comparisons to understand the biological changes occurring on a broader level both across lifespan and between age periods, and we found the regulation of peptidyl‐tyrosine phosphorylation to be the top pathway of CSF aging in vervets (Figure 2D). A PPI network was created using hits from the linear model of general aging, with linker genes to visualize mutual interactions between hits that otherwise may not have one based on the markers entered. Although the whole network was most represented by “cytokine‐cytokine receptor interaction” the six clusters that were identified by the ReactomeFI program had overrepresented pathways such as “positive regulation of cell population proliferation,” “dendrite self‐avoidance,” “negative regulation of endopeptidase activity,” “positive regulation of leukocyte chemotaxis,” “cytokine‐cytokine receptor interaction,” and “positive regulation of lysosome organization” (Figure 2E).

When looking at sex differences with aging, 39 markers showed effects (Figure 2F,G); no marker passed multiple comparisons. Several members of the complement cascade were hits, including C1QA, C3, C9, CFI, and SERPING1. C1QA, an initiating member of the classical complement cascade, was the top hit (*p* = 0.0003, FDR Q = 0.137) and increased in males with age but decreased in females. Of interest, all significant complement factors exhibited the same relationship with age and sex. As expected, pathway analysis revealed “regulation of complement cascade” to be the most over‐represented pathway (Figure 2H). After discovering biomarkers that linearly change with age, we were curious to see if there were markers that demonstrate notable non‐linearity, and if so, were there others like them. These proteins may not be hits in the linear model; however, drastic swings and affected pathways would present biological significance. Unsupervised hierarchical clustering of protein z‐scores was done to identify eight clusters of CSF proteins with similar trajectories (Figure 2I). It is notable that although some clusters display relatively linear changes, several demonstrated wave‐like movement. For example, cluster 3 was enriched for the “complement cascade” and had a linear‐like decline with age, whereas cluster 4 shows peaks around the late teens and mid‐twenties and was enriched for “leukocyte migration.” Similar trajectories to cluster 4 are seen in clusters 5, 6, and 7, which are enriched for “regulation of cytokine‐mediated signaling pathway,” “cell chemotaxis,” and “peptidyl‐tyrosine phosphorylation,” respectively.

#### 3.2.2 Investigating FGF18 and TROY's relationship with aging and Aβ

**FIGURE 3:**
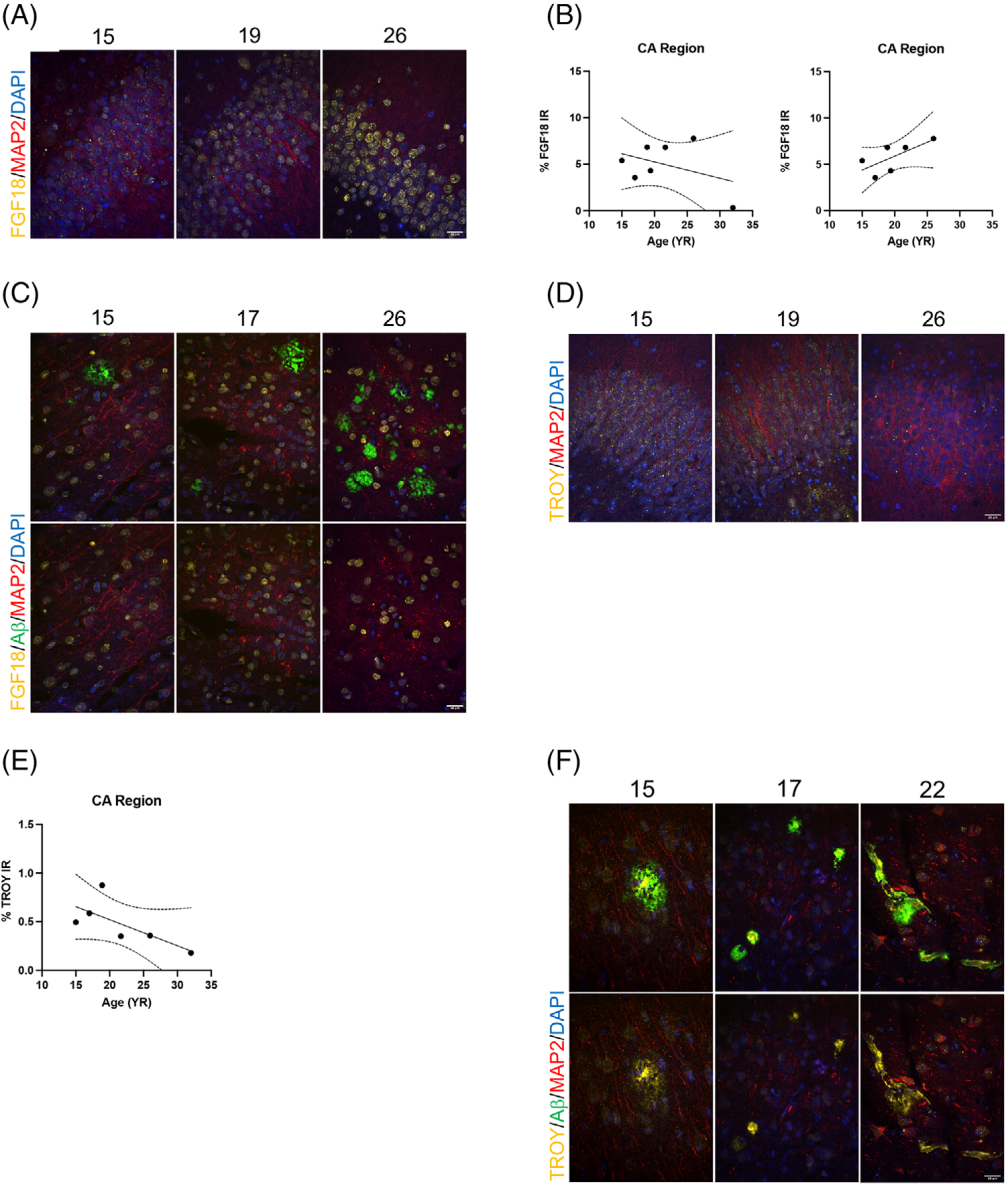
(A) Images of FGF18 in the stratum pyramidale layer of the CA region in three vervets with age. (B) Linear regression analysis of FGF18 IR mostly increasing with age including (*p* = 0.37) and excluding (*p* = 0.12) a 32 YO vervet (right). Dashed lines indicate 95% confidence band. (C) Images of FGF18 in the presence of Aβ. Bottom row images do not include Aβ (green) to show FGF18 IR beneath. (D) Images of TROY in the stratum pyramidale layer of the CA region in three vervets with age. (E) Linear regression analysis showing TROY IR decreasing with age (*p* = 0.12). Dashed lines indicate 95% confidence band. (F) Images of elevated TROY in the presence of Aβ plaque and vascular amyloid. Bottom row images do not include Aβ (green) to show TROY IR beneath. Scale bars: 20 μm.

To further explore the relationships of FGF18 and TROY with aging in the brain, we conducted immunofluorescent studies. To assess whether the elevation of FGF18 in the CSF with age could also be found in the hippocampus, we compared FGF18 IR in the stratum pyramidale layer of the CA region in seven vervets ranging from 15 to 32 YO (Figure 3A,B). FGF18 expression was observed in neurons as well as other unidentified cells and was heterogenous, it was found punctated, diffusely distributed, concentrated in one area, and lining the membrane of cells. IR generally increased with age; however, notably low levels were found in a 32 YO vervet whose FGF18 IR resembled that of the 15 YO vervet. Linear regression analysis showed an overall decrease in FGF18 IR (*p* = 0.37); however removing the 32 YO vervet as an outlier from the analysis better reflected our CSF results, as we saw IR then increase with age (*p* = 0.12; Figure 3B). Levels of FGF18 in neurons, visualized using MAP2, did not appear to be affected by Aβ pathology (Figure 3C). We next studied TROY, which strongly declined in CSF with age (Figure 2A). Looking in the same area of the hippocampus of six vervets ranging from 15 to 32 YO, TROY IR decreased with age (*p* = 0.12) and was observed mostly punctated in neurons (Figure 3D,E). Surprisingly, we consistently found TROY to be highly colocalized with fibrillar, compact, and cored Aβ plaques and vascular amyloid (Figure 3F). In addition, we did not find that this Aβ‐associated TROY activity to differ with age.

#### 3.2.3 CSF biomarkers of cognition (ORT score)

**FIGURE 4:**
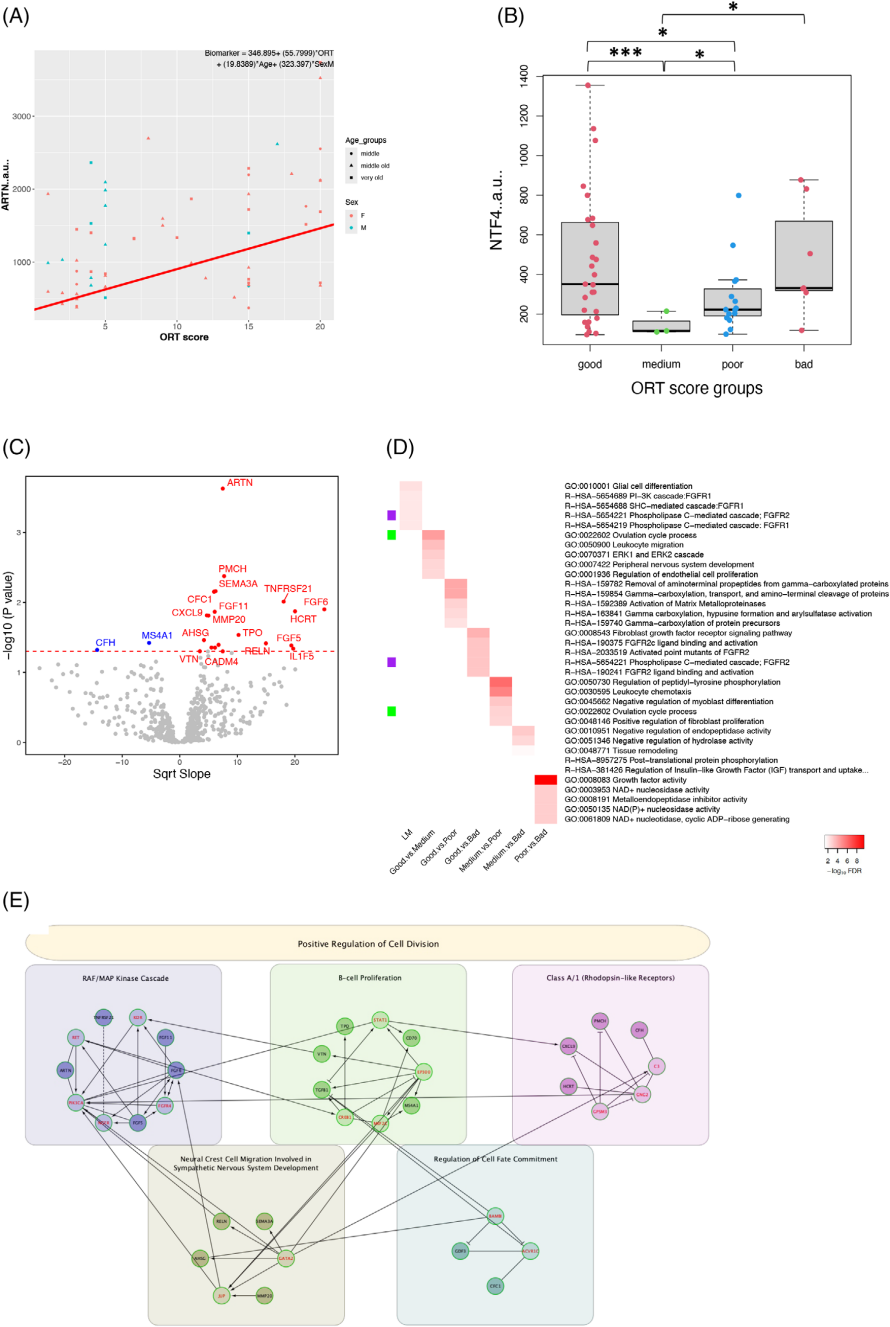
(A) Top hit of the linear model of ORT score, ARTN. A higher ORT score indicates worse performance. (B) Box plot of ORT score group comparisons for NTF4, which had highly significant differences between group comparisons. (C) Volcano plot of the linear model of ORT. Red dots represent proteins that increased with ORT score, indicating association with poor cognition. (D) Heatmap of top five pathways for hits in each ORT analysis. (E) PPI network of the hits from the linear model of ORT. Arbitrary units (a.u.).*p < 0.05,***p < 0.001.

Cognitive performance was measured in the vervets using ORT, a test of spatial and episodic memory where a higher score equals worse performance. A linear model, as well as ANOVAs and *t*‐tests between ORT score groups, was performed to find CSF biomarkers that show effects with cognition. ORT score groups consisted of “Good” (0–5), “Medium” (6–10), “Poor” (11–15), and “Bad” (16–20). The linear model revealed 22 proteins to significantly change with ORT score (Figure 4A,C). Artemin (ARTN), a member of the glial cell line–derived neurotrophic factor (GDNF) family, was the top hit (*p* = 0.0002, FDR Q = 0.126) and increased with ORT score (Figure 4A). Neurotrophin‐4 (NTF‐4), another neurotrophic factor that was part of the same family that includes GDNF, brain‐derived growth factor, and nerve growth factor, mediates neuronal growth and survival and showed strong ORT score group‐specific effects (Figure 4B). There were significant reductions in the medium and poor groups compared to the good group (*p* = 0.0003, FDR Q = 0.057; *p* = 0.031, FDR Q = 0.985) and significant increases in the poor and bad groups compared to the medium group (*p* = 0.035, FDR Q = 0.98; *p* = 0.024, FDR Q = 0.988). Pathway analysis of the linear hits and the ORT group comparisons revealed broader biological processes associated with cognition in the CSF (Figure 4D). The top pathway of the linear model was “glial cell differentiation.” A PPI network was created, and five clusters were identified using the ReactomeFI program (Figure 4E). The top pathway of the whole network was “positive regulation of cell division” and clusters were represented by “RAF/MAP kinase cascade,” “B‐cell proliferation,” “class A/1 (rhodopsin‐like receptors),” “neural crest cell migration involved in sympathetic nervous system development,” and “regulation of cell fate commitment.” Although direct interaction between hits was limited, linker genes provided abundant connections, both between hits and other linker genes, suggesting the presence of underlying interconnectivity among the hits. In addition, when looking at linear associations with TSP, ARTN was again the top hit (*p* = 3.18E‐5, FDR Q = 0.017, Bonferroni Q = 0.017), and similar to ORT score, increased with worsening percentages of overall success (Figure S1A). Pathway analysis of the hits revealed “growth factor receptor binding” to be the most highly implicated, followed by the complement cascade (Figure S1B).

#### 3.2.4 Investigating Artemin's relationship with cognition and AD pathology

**FIGURE 5:**
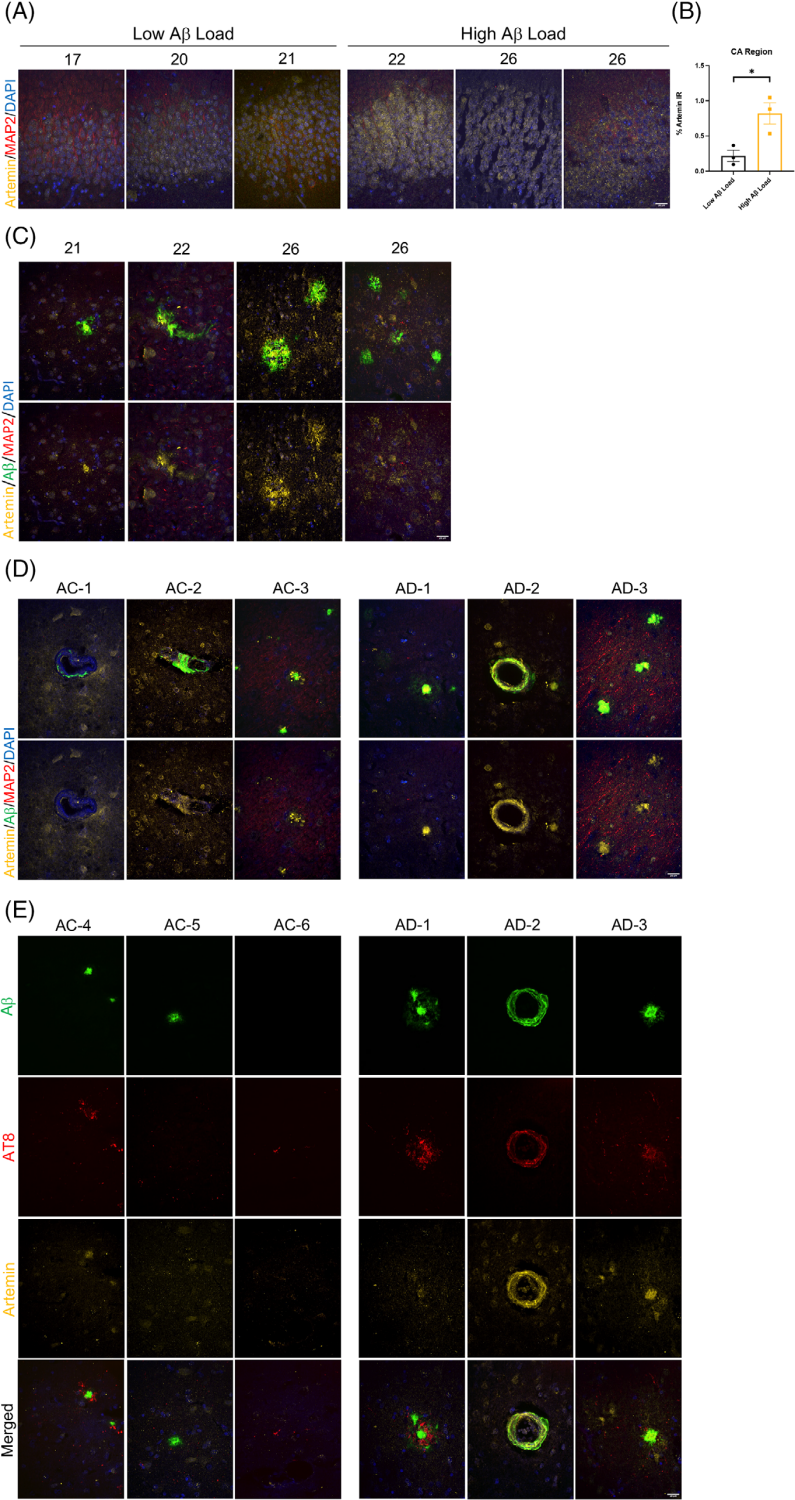
(A) Images from the stratum pyramidale layer of the CA region in six aged vervets divided into two groups based on Aβ burden. (B) Two‐tailed Student's *t*‐test comparing the percent immunoreactivity of ARTN in the low versus high Aβ groups. Error bars represent SEM. ARTN was significantly higher in the high Aβ group (*p* = 0.02). (C) Images from the TC/HC region of aged vervets showing an increase in ARTN expression at the site of Aβ pathology, including vascular amyloid. (D) Images of increased ARTN expression with Aβ pathology in the parietal lobe of three human AD patients and three aged, non‐demented controls (AC). (E) Images from the TC/HC region of the same three human AD patients and a different set of 3 AC patients. AT8 was used to show ARTN's relationship with neurodegeneration. Scale bars: 20 μm.

To further investigate ARTN's relationship with cognition and potentially AD pathology, immunofluorescent staining was done in aged vervets as well as human AD patients and aged, non‐demented controls (AC). Using the stratum pyramidale layer of the CA region as a structural landmark, we assessed differences in ARTN IR between three aged vervets with low Aβ IR, as observed through our initial IHC studies, and three aged vervets with high Aβ burden (Figure 5A). Of interest, ARTN was significantly elevated (*p* = 0.02) in the high Aβ group (Figure 5B), adding to our finding of increased levels with poorer ORT scores (Figure 4B). In the TC/HC region of the same vervets, we consistently observed ARTN expression at the site of compact and/or cored Aβ plaques as well as vascular amyloid but rarely with diffuse Aβ, indicating a relationship with advanced AD pathology (Figure 5C). Positive staining was confirmed using adjacent sections with and without secondary antibody (Figure S2A). Furthermore, ARTN was also found spread across extracellular matrices and appeared in a punctated pattern in the soma of MAP2‐positive neurons (Figure 5C).

To investigate how these findings translated to humans, parietal sections from three AD patients and three AC cases were also stained (Figure 5D). ARTN was found in both AC and AD cases but was especially abundant with vascular amyloid and what appeared to be neuritic‐cored plaques in AD patients, indicating its expression is proportional to the degree of pathology. Case AC‐2 presented a unique finding of concentrated areas of MAP2 fibers, most likely dendrites, with severe ARTN staining of the matrix surrounding the area (Figure S2B). This co‐localization was observed repeatedly in this sample; however, similar MAP2 IR was not seen in other cases. Future work should explore this finding, which we believe may be a response to an unidentified stressor on the neuron. To further investigate our findings, we looked at human TC/HC sections and used AT8 as a marker of neurodegeneration and to confirm neuritic plaques (Figure 4E). AD cases 1, 2, and 3 as well as AC‐4 demonstrate ARTN's presence in AT8‐positive Aβ plaques, whereas in AC‐5 we saw no ARTN signal in an AT8‐negative Aβ plaque. However, this may also be due to the plaque being more fibrillar rather than compact or cored. Of interest, AC‐6 presented a case of a neurite with positive AT‐8 staining in the absence of Aβ and without ARTN signal. Additional staining with GFAP was done in adjacent TC/HC sections of the AD brains to determine whether the ARTN observed with this pathology came from astrocytes (Figure S2C); however, with minimal ARTN signal found in their soma, further work is needed to explore other possibilities, such as microglia or leakage from an adjacent neuron.

### 3.3 Plasma biomarkers

#### 3.3.1 Plasma biomarkers of aging

**FIGURE 6:**
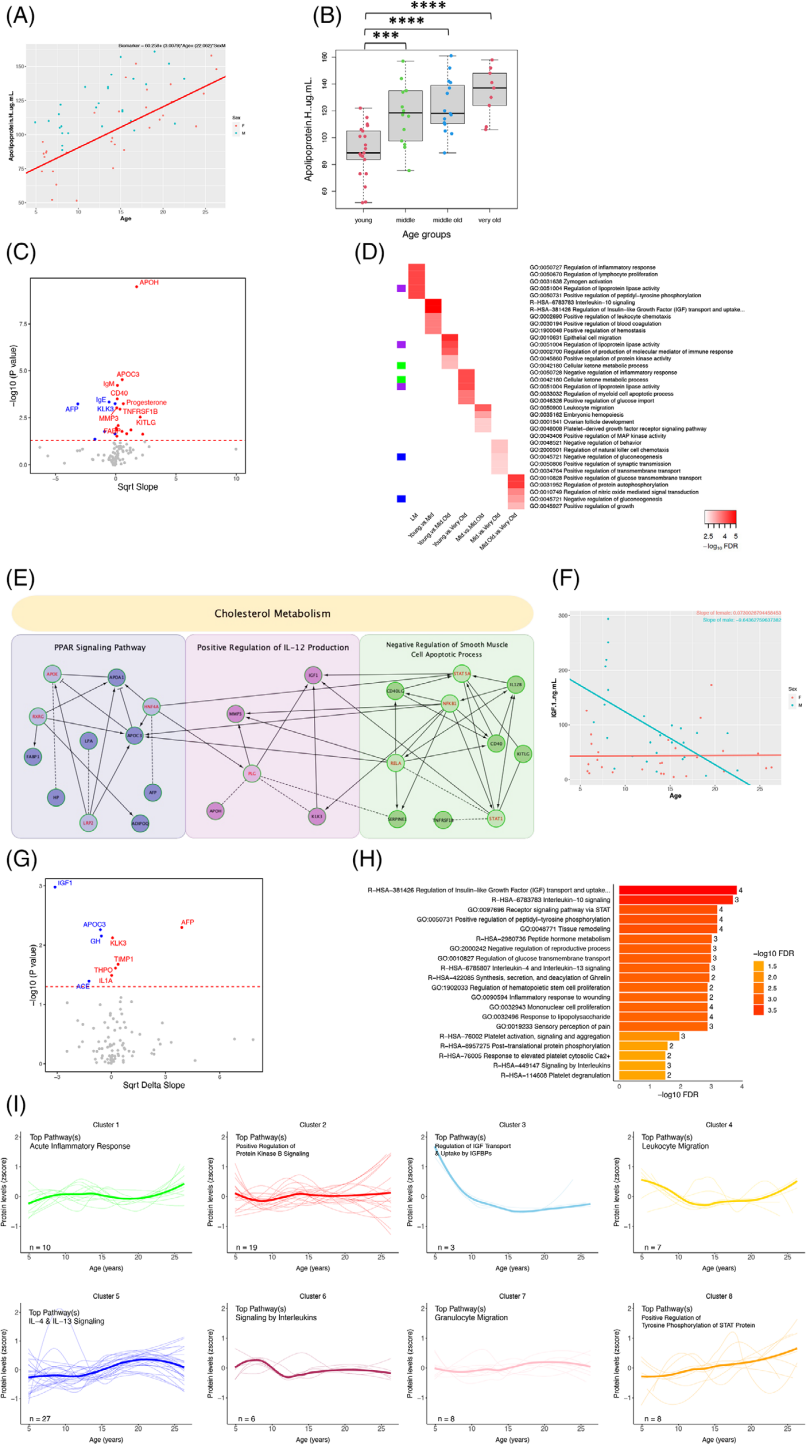
(A) Linear regression of the top hit with aging, adjusted for sex. (B) Box plot of apoH. Asterisks indicate *p*‐value significance level (***p < 0.001, ****p < 0.0001). (C) Volcano plot of proteins with aging showing square root transformed slope and –log_10_ *p*‐value. (D) Heatmap of top five pathways based on hits from each analysis. Matching sidebar colors indicate overlapping pathways. (E) PPI network based on hits from linear model of general aging with linker genes (red labels) included. (F) Top hit from sex differences with aging analysis. (G) Volcano plot of proteins showing sex differences with aging. (H) Bar plot of top 20 pathways implicated in sex‐specific aging. (I) LOESS trajectories of z‐scores for eight clusters of plasma proteins. Top pathway and number of proteins in each cluster are indicated.

In a linear model of aging, 22 biomarkers demonstrated significant effects (nominal *p* < 0.05, Figure 6A,C), and of these, 17 remained significant after FDR corrections (Q < 0.1) and 9 after Bonferroni corrections (Q < 0.1). The top hit was apolipoprotein H (apoH; *p* = 3.30E‐10, FDR Q = 3.27E‐8, Bonferroni Q = 3.27E‐8) (Figure 6A), a protein involved in cholesterol and lipid metabolism. Looking at differences in apoH levels between age groups (Figure 6B) showed significant, step‐like increases in the middle (*p* = 0.0009, FDR Q = 0.077, Bonferroni Q = 0.077), middle‐old (*p* = 2.19E‐5, FDR Q = 0.002, Bonferroni Q = 0.002), and very‐old (*p* = 2.41E‐5, FDR Q = 0.002, Bonferroni Q = 0.002) groups when compared to the young group. There were non‐significant differences between the middle, middle‐old, and very‐old groups, although there was a near‐significant elevation in levels in the very‐old vervets compared to the middle‐aged ones (*p* = 0.077). Pathway analyses of the linear model hits revealed the regulation of inflammatory response and lipoprotein lipase activity in plasma to be processes highly implicated in aging in vervets (Figure 6D). Interactions between the same hits were visualized using ReactomeFl; clusters and their top pathways or biological processes are shown as well as for the whole network (Figure 6E).

We further examined whether any of the biomarkers demonstrated sex‐specific aging effects and found this to be the case with nine markers (Figure 6F,G). In addition to showing effects with general aging, insulin‐like growth factor‐1 (IGF‐1), a key regulator of glucose metabolism and growth, showed the strongest difference between sexes with linear aging (*p* = 0.001, FDR Q = 0.05, Bonferroni Q = 0.1004) wherein levels were initially greater in males but decreased with age, whereas females exhibited a more consistent relationship with age (Figure 6F). Furthermore, pathway analysis of proteins with significant age‐associated sex‐differences revealed “Regulation of insulin‐like growth factor transport and uptake by insulin‐like growth factor binding proteins” to be a top pathway (Figure 6H). We again wanted to find clusters of proteins that move similarly with age across the lifespan, and eight were created ranging from 3 to 27 proteins (Figure 6I). Cluster 1, for example, contains 10 proteins enriched for acute inflammatory response, which move in a wave‐like fashion across the lifespan of the vervets. Of interest, cluster 5, which was enriched for IL‐4 and IL‐13 signaling, had relatively unremarkable changes until the early teenage years, after which there was notable rise in levels followed by a slight reduction in the oldest years. Furthermore, the clusters with more pronounced non‐linearity tended to be immune related.

#### 3.3.2 Plasma biomarkers of cognition (ORT score)

**FIGURE 7:**
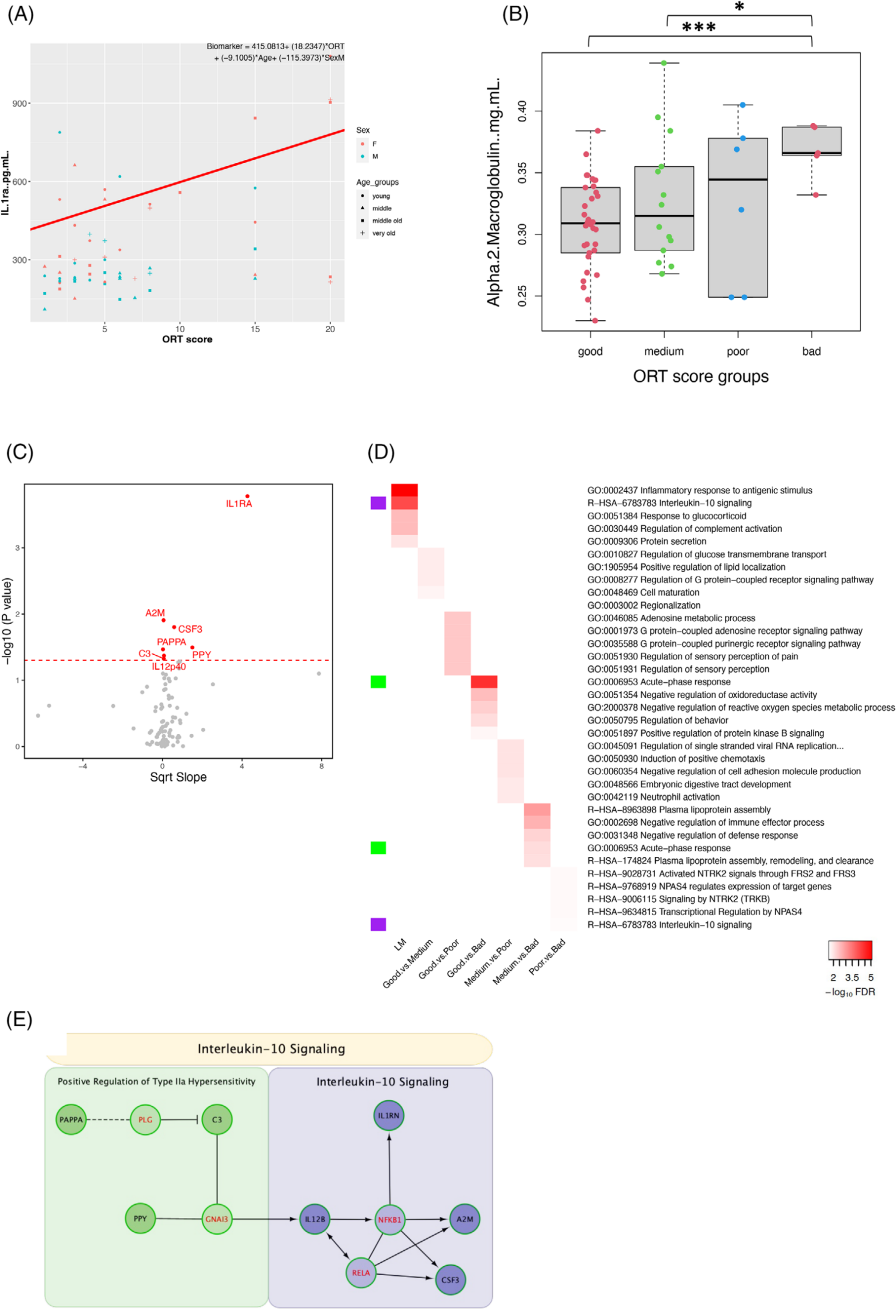
(A) Top hit of the linear model of ORT score adjusted for sex. (B) Box plot of ORT score group comparisons. A2M had the strongest effect with group comparisons among all proteins. (C) Volcano plot of the linear model of ORT. (D) Heatmap of top five pathways for hits in each ORT analysis. (E) PPI network of the hits from the linear model of ORT. *p < 0.05,***p < 0.001.

When searching for plasma biomarkers that associated linearly with cognitive performance, seven proteins showed significant effects, of which IL‐1 receptor antagonist (IL‐1ra) was the only marker to pass multiple comparisons (*p* = 0.0002, FDR Q = 0.016, Bonferroni Q = 0.016; Figure 7A,C). IL‐1ra, which works to oppose IL‐1 activity, increased with ORT score, demonstrating an inverse relationship with cognitive performance (Figure 7A). All hits from the linear model increased with ORT score (Figure 7C). Alpha‐2 macroglobulin (A2M) was the next top hit in the linear model and had the strongest effect of any ORT score group comparisons between the markers. Levels of A2M, a pan‐protease inhibitor, were significantly elevated in the bad group compared to both the good (*p* = 0.0009, FDR Q = 0.075, Bonferroni Q = 0.075; Figure 7B) and medium groups (*p* = 0.033, FDR Q = 0.547; Figure 7B). After performing pathway analysis, we found “Inflammatory response to antigenic stimulus” to be the top biological process in the linear model (Figure 7D). Linear hits were divided into two clusters in ReactomeFI, with one cluster involving “Positive regulation of type IIa hypersensitivity” and the other “Interleukin‐10 signaling,” which was also the top pathway of the network. Linker genes NFKB1 and RELA provided many interactions between IL‐12p40, A2M, and CSF3, and have been heavily implicated in aging and AD pathogenesis themselves (Figure 7E).^33^ When looking at TSP, IL‐1ra was again the top hit (*p* = 0.0009, FDR Q = 0.083, Bonferroni Q = 0.083) and inversely associated with overall cognitive performance (Figure S1C). It is notable that as found in our analysis of CSF hits with TSP, “growth factor receptor binding” was also the top pathway with our plasma hits (Figure S1D).

#### 3.3.3 Plasma biomarkers associated with CSF Aβ

**FIGURE 8:**
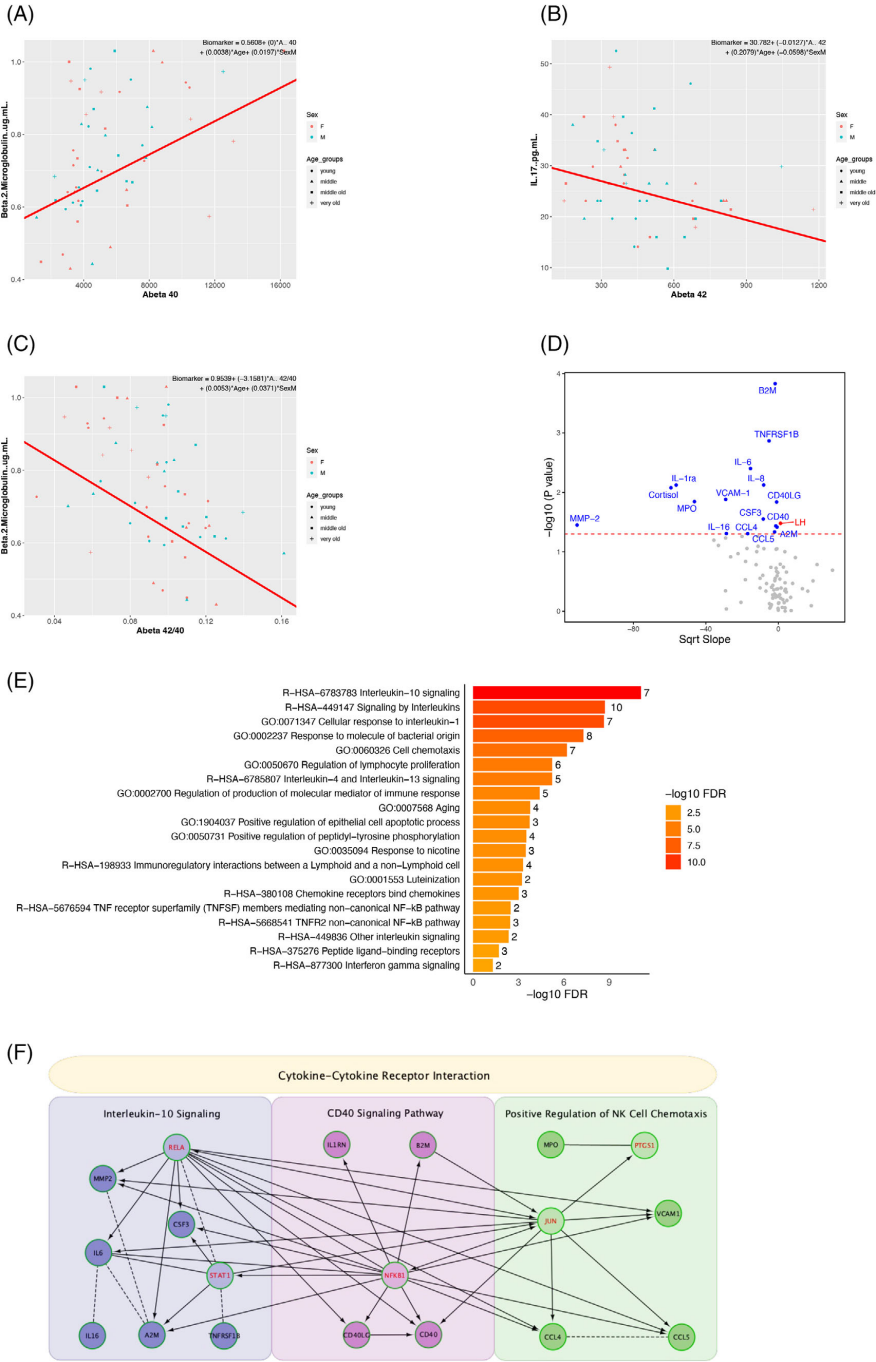
(A–C) The plasma proteins most significantly associated with CSF Aβ40 (A), Aβ42 (B), and Aβ42/40 (C), respectively. (D) Volcano plot of linear model of Aβ42/40. Blue dots indicate proteins that decreased as the ratio increased. (E) Top 20 pathways from hits of the Aβ42/40 linear model. (F) PPI network of Aβ42/40 hits.

We also measured levels of CSF Aβ40 and 42 in the same animals to see if there were plasma biomarkers that were significantly associated. We were particularly interested in finding biomarkers associated with the ratio of Aβ 42/40 given its greater predictive value than Aβ42 or Aβ40 alone and its strong correlation with amyloid PET.^34^, ^35^ Beta 2‐microglobulin (B2M) was the top hit when looking at CSF Aβ40 (Figure 8A) and correlated positively with levels (*p* = 0.001, FDR Q = 0.035, Bonferroni Q = 0.105), whereas IL‐17 had the strongest association with CSF Aβ42 (*p* = 0.028, FDR Q = 0.887) and demonstrated a negative correlation (Figure 8B).

When looking at the ratio of CSF Aβ42/40, we found 17 plasma markers to have significant effects (Figure 8C,D), of which B2M, TNFRSF1B, and IL‐6 remained after FDR correction, with B2M having the strongest association (*p* = 0.0001, FDR Q = 0.005, Bonferroni Q = 0.015). A lower CSF Aβ42/40 ratio is indicative of a poorer clinical status, and we found B2M, involved in the innate immune system, to increase as the ratio decreased (Figure 8C). Luteinizing hormone (LH) was the only hit to decrease as the ratio of Aβ increased (Figure 8D). Several of the hits in this analysis belonged to the interleukin family and the most overrepresented pathway was “Interleukin‐10 signaling” (Figure 8E). Of interest, RELA and NFKB1 were again highly connected linker genes in the PPI of the linear model. Although the “cytokine‐cytokine receptor interaction” was enriched among the whole network, the three clusters were enriched for “interleukin‐10 signaling,” “positive regulation of NK cell chemotaxis,” and “CD40 signaling pathway,” which is involved in microglial activation (Figure 8F).

## 4 DISCUSSION

Previous work has shown the potential for vervets to serve as models of AD and age‐related cognitive decline through transcriptional profile comparisons, as age‐related changes in gene expression profiles were comparable between AD patients and vervets and both profiles were enriched with inflammatory cytokine genes.^18^ In addition to providing further evidence of the natural, age‐related increases in Aβ plaque burden in both the FC and TC/HC regions of the vervet, we also used proteomics to explore the relationship between changes in plasma and CSF proteins as a function of aging, sex, cognitive decline, and levels of CSF Aβ (a well‐defined AD biomarker).

CSF analysis revealed a substantial number of analytes linearly correlated with age; many of these were related to regulating peptidyl‐tyrosine phosphorylation. Of interest, altered protein tyrosine phosphorylation has been previously suggested to be involved in AD pathogenesis.^36^ Of the markers implicated in general aging, several belonged to the fibroblast growth factor (FGF18, FGF3, FGF20, FGF14), tumor necrosis factor (TNFRSF19, TNFSF4, TNFRSF9, TNFSF10), or interleukin (IL‐20, IL‐1F9, IL‐3, IL‐6R, IL‐12A, IL‐1B) families. Deeper investigation of FGF18 revealed a non‐significant increase in IR in the stratum pyramidale layer of the CA region in the hippocampus except for a 32 YO vervet near the end of its lifespan having notably reduced IR with strong puncta. No co‐localization between FGF18 and Aβ was observed, which may be explained by the ability of FGF18 to reduce Aβ accumulation.^37^ TROY, in addition to being found in the soma of MAP2‐positive neurons, demonstrated a surprisingly high degree of co‐localization with Aβ including fibrillar, compact, and cored plaques, and vascular amyloid, and this association appeared equally strong across age. TROY is part of the tumor necrosis family and has been implicated heavily in glioblastoma, where it correlates inversely with survival and is overexpressed in tumors, stimulating cell migration and invasion.^29^ In addition, it regulates vascular development in the brain, plays a role in the disruption of the structure/function of the blood–brain barrier during aging,^30^ mediates myelination,^38^ and contributes to genetic susceptibility to vascular dementia.^39^ Here we add to these previous findings and, to the best of our knowledge, show for the first time TROY's co‐localization with AD pathology, which we believe is an inflammatory response to Aβ toxicity. Analysis of TROY IR in the hippocampus paralleled our findings of declining levels in CSF with age. Several complement proteins were related to general aging (C3, SERPING1, CFP). C3 and SERPING1, a C1 inhibitor, also showed sex differences with aging, as did C1QA, CFI, and C9, where levels declined in females and increased in males. The complement cascade is responsible for a highly effective, choreographed innate immune response that removes damaged cells, tags synapses for destruction, aids wound and injury repair, and fights infections. C1Q is an initiating member of the classical complement cascade and consists of sub‐components “A” and “B.” In the CNS, complement proteins such as C1Q mediate synaptic pruning and neurodegeneration; levels of C1Q have been found to dramatically increase with normal aging in the brain, especially in the hippocampus.^40^ Further studies are needed on sex‐specific differences in C1QA with aging, but several complement proteins in the CSF have been found to be lower in women (C3, SERPING1, and CFI).^41^, ^42^ Our results support these findings of sex differences in the complement cascade, and we add them in the context of a model known to develop AD amyloid deposition naturally with age. To capture the total complexity of the relationship of CSF biomarkers with age, we identified clusters of proteins with similar global trajectories, and found some to demonstrate especially non‐linear movements with multiple waves across lifespan. Furthermore, these clusters happened to be involved in pathways overrepresented for processes related to immune signaling and suggest similarities in the biological processes occurring at these distinct age periods that repeatedly drive elevations of these proteins. Although there were no overlapping hits between the aging and cognition analyses, members of the fibroblast growth factor family were again implicated in cognitive performance. FGF proteins have diverse functions including cell survival, migration, differentiation, and proliferation; they also regulate central nervous system development, such as synaptogenesis and axon guidance. FGF treatment has restored neurogenesis and hippocampal functions in aged mice and mouse models for AD, and therefore, may have therapeutic abilities.^43^ Some members of the FGF family, such as FGF21 and 23, have previously shown an inverse correlation with cognition, which is similar to our evidence that FGF 5, 6, and 11 increase with ORT score.^44^, ^45^ The top CSF hit of the linear ORT analyses was vascular‐derived neurotrophic factor, ARTN, whose effects include supporting growth and survival of peripheral and central neurons (especially sympathetic neurons) and repairing nerve injury.^46^, ^47^ In addition, ARTN has chaperone activities against pathological AD proteins, such as blocking fibrillization of α‐synuclein and tau in vitro.^48^, ^49^ Although further research is needed on the direct relationship between CSF levels of ARTN and cognition, its close relative GDNF has been shown to be essential for cognition and hippocampal integrity, as deletions of the *GDNF* gene or its receptor impairs learning and memory in rodent models.^50^ In addition, a clinical study indicated that CSF levels of GDNF increased in early stages of AD and suggested that this may be due to an adaptive process to enhance neurotrophic support in the early stages of disease.^51^ We add, for the first time, that ARTN levels in CSF not only inversely correlate with cognition but that ARTN IR can also be found elevated in the CA region of aged vervets with high Aβ burden. Furthermore, ARTN co‐localizes with vascular amyloid as well as compact and neuritic‐cored Aβ plaques in vervets and humans. When including AT8, we found cases where ARTN was absent in the presence of Aβ but without AT8 and with AT8 but without Aβ; however, in an AC case as well as AD cases we saw ARTN IR in AT8‐positive Aβ plaques. These results suggest that ARTN colocalization may require “two hits”: amyloid deposition and degenerating plaque‐associated neurites. Although we cannot define the cell type from which ARTN is released, it is possible that ARTN is released in an attempt to combat pathological stress and promote cell survival. However, further studies with larger sample sizes should examine when ARTN expression is upregulated and if this response is exhausted.

Plasma biomarkers for many disorders have garnered increasing interest due to their ease of clinical access, and particular attention has been placed on finding markers of aging and AD. Fortunately, our study revealed multiple markers that may help diagnose and/or inform the mechanisms behind these conditions. ApoH demonstrated a highly significant linear increase with age as well as effects between increasingly old age groups. It has roles in apoptosis, coagulation, inflammation, defense against bacterial infections, angiogenesis, and atherogenesis, and is believed to play a role in neurodegenerative diseases.^52^, ^53^, ^54^ For example, apoH has been linked to general aging, cognitive aging, mild cognitive impairment (MCI), and AD.^52^, ^55^, ^56^, ^57^ However, it was not the only lipoprotein to show aging effects. Apolipoprotein C3 (apoC3), apolipoprotein A1 (apoA1), and lipoprotein A (LPA) all increased significantly with age, and thus top pathways of the linear model were “regulation of lipoprotein lipase activity” and “regulation of inflammatory response.” This finding confirms similar reports of apolipoproteins A1, A2, B, C3, and H declining in a cohort consisting of middle‐aged to very‐old human adults (56–105 YO).^58^ Although these findings conflict with some of the increases in apolipoproteins we report here, this is most likely because our study looked at the total lifespan of vervets and the mentioned study looked only at the second half of the human life span. In addition, survivorship bias may be at play. Several hits from the general aging model also showed sex‐specific effects, including IGF‐1, alpha‐fetoprotein, apoC3, and prostate specific antigen. IGF‐1, in particular, plays an important role in metabolism that makes it highly implicated in aging and longevity. It has been shown to have sex‐specific differences including a greater decline in men with age,^59^ as we observed with male vervets, and consequently has differential disease risks, such as reduced bone density and increased fracture risk in women with low levels, and lower mortality and insulin resistance in female mice with a heterozygous knockout for IGF‐1 receptor.^60^, ^61^ In addition, because it is actively transported across the blood–brain barrier, it is believed to have neuromodulatory effects. For example, IGF‐1 signaling is impaired in AD and affects the clearance of Aβ in the brain.^62^ It is interesting to note that the regulation of peptidyl‐tyrosine phosphorylation was a top biological process of aging in both plasma and CSF, indicating parallels in the mechanisms that drive aging between the two systems. When looking for plasma biomarkers that associated significantly with cognitive performance (ORT), we found IL‐1ra to be a top hit. The balance between IL‐1 and IL‐1ra can determine the severity of many diseases, primarily those with epithelial or endothelial cell origin.^63^ Little research has been done to specifically isolate the effects of plasma IL‐1ra levels on cognition in patients with AD. However, in a study on mucopolysaccharidosis IIIA, a disease also involving Aβ, IL‐1ra gene therapy was able to prevent behavioral abnormalities and cognitive decline caused by the neuroinflammatory effects of IL‐1.^64^ Other studies have shown that elevated levels of serum IL‐1ra were significantly, negatively associated with cognition in schizophrenic, bipolar, and healthy patients,^65^, ^66^ paralleling our results. It is notable that previous work looking at longitudinal changes in the blood transcriptome of vervets under psychosocial stress found IL‐1ra to decrease over time.^19^ In addition, we found A2M to have strong effects between ORT score groups, which is consistent with previous findings in humans. A2M has long been known to be genetically involved with AD, to the extent that it is recognized as a LOAD gene.^67^ In AD brains, A2M has been shown to localize with diffuse amyloid plaque and affect the degradation of Aβ, as well as mediate its clearance.^68^ Results from other studies show that plasma levels are significantly elevated in MCI and AD patients compared to controls,^69^ and here we find a similar relationship between levels and cognition. Plasma B2M demonstrated a strong association with not only CSF Aβ40 but the ratio of Aβ42/40, which is more predictive of brain pathology than either Aβ species alone. B2M plays a role in antigen presentation through its composition of the light chain of MHC I.^70^, ^71^ It has been shown to increase with age in the blood and CSF of humans and in the blood of mice, as well as the hippocampus, and has been determined to promote age‐related cognitive decline and impaired neurogenesis. This pro‐aging factor has also been shown to be elevated in the blood and CSF of AD patients compared to controls and to be predictive of risk of acute ischemic stroke.^71^, ^72^, ^73^, ^74^, ^75^ Additionally, B2M aggravates Aβ pathology, and coaggregation is essential for Aβ neurotoxicity in an AD mouse model.^76^ Several hits in the linear ORT score analysis overlapped with that of Aβ42/40, potentially identifying biomarkers associated with AD‐related cognitive impairment. These hits include the known AD marker A2M, as well as IL‐1ra and granulocyte colony stimulating factor 3. Furthermore, many hits in the ORT and Aβ42/40 analyses were immune markers, and a top overlapping pathway between them was “interleukin‐10 signaling.” We also found TNFRSF1B, CD40, and CD40 Ligand (CD40LG) to show effects with both linear aging and Aβ42/40, with the regulation of lymphocyte proliferation and peptidyl‐tyrosine phosphorylation being shared overrepresented pathways. The CD40‐CD40LG dyad is part of the tumor necrosis factor family and has been associated repeatedly with AD.^77^ It mediates several immune responses, including Aβ‐induced microglial activation, which disturbs genes regulating APP and tau phosphorylation, and as a result, causes neurofibrillary tangles and plaques.^78^ CD40 and its ligand have been found to be elevated in the plasma of AD patients and CD40 expression is higher in the blood of older adults.^79^, ^80^ We also report a significant increase in CD40 in vervets with age, although we found CD40LG to decrease. Both CD40 and CD40LG were inversely associated with the ratio of Aβ, pointing to similar findings of elevated levels in AD patients.

Although this study is exploratory, it is the first report to our knowledge of plasma and CSF proteomics that revealed biomarkers of aging and cognitive decline in vervets. In addition, we identified some hits that have not been associated previously with aging or cognitive decline. However, although ORT allowed us to assess cognitive health, its reliability as a single measure is limited. Another limitation of this work was the inability to execute the initial longitudinal design of the CSF study, which future work would benefit from. In addition, linker genes in the PPI networks should be included in future studies, given their high level of interconnectedness with the top hits. Finally, apart from a study involving the use of an environmental toxin,^81^ vervets develop minimal neurofibrillary tangles and tau pathology.^82^ Therefore, such analyses were not included, and our results are more relevant to earlier stages of AD than later.

With an aging population at risk of cognitive decline and AD, the utility of biomarkers is ever‐increasing. Here we showed that vervets have translational value. In addition to confirming their natural development of Aβ pathology in both the FC and TC/HC regions, through proteomics we discovered numerous parallels between our significant aging and cognition markers in vervets and humans, which allowed us to show for the first time that vervets have highly similar peripheral and central responses to aging and cognitive decline. We also provided a basis for investigation of markers not previously shown to associate with these variables, such as ARTN, which we discovered has similar pathological responses to cored Aβ plaques in both vervets and humans, further proving the translatability of vervets. An especially valuable component of this study was finding plasma biomarkers associated with CSF Aβ levels, a first in vervets.

## CONFLICT OF INTEREST STATEMENT

R.M.P. is the Scientific Officer for the Behavioural Science Foundation, a not‐for‐profit research foundation. C.A.L. serves as a paid scientific advisor for Acumen Pharmaceuticals, ADvantage Therapeutics, Alnylam Pharmaceuticals, Apellis Pharmaceuticals, Cyclo Therapeutics, Merck, Novo Nordisk, and Sanofi‐Genzyme, and receives research funding from the National Institutes of Health (NIH), NASA, and the Cure Alzheimer's Fund. All other authors have no conflicts to report. Author disclosures are available in the Supporting Information.

## CONSENT STATEMENT

Human subjects and/or their families provided written informed consent for brain tissue donation.

## Supporting information

Supporting Information

Supporting Information

Supporting Information

Supporting Information

Supporting Information

Supporting Information

Supporting Information

## References

[1] Alzheimer's: Facts, Figures & Stats n.d . Accessed February 12, 2024. https://www.brightfocus.org/alzheimers/article/alzheimers‐disease‐facts‐figures

[2] Mendez MF . Early‐Onset Alzheimer disease. Neurol Clin. 2017;35:263‐281. 28410659 10.1016/j.ncl.2017.01.005 PMC5407192

[3] Guerreiro R , Bras J . The age factor in Alzheimer's disease. Genome Med. 2015;7:106. 26482651 10.1186/s13073-015-0232-5 PMC4617238

[4] Sperling RA , Aisen PS , Beckett LA , et al. Toward defining the preclinical stages of Alzheimer's disease: recommendations from the National Institute on Aging‐Alzheimer's Association workgroups on diagnostic guidelines for Alzheimer's disease. Alzheimers Dement. 2011;7:280‐292. 21514248 10.1016/j.jalz.2011.03.003 PMC3220946

[5] Medical Tests for Diagnosing Alzheimer's . Alzheimer's Disease and Dementia n.d. Accessed February 12, 2024. https://alz.org/alzheimers‐dementia/diagnosis/medical_tests

[6] Alzheimer's disease . Mayo Clinic 2023. Accessed February 12, 2024. https://www.mayoclinic.org/diseases‐conditions/alzheimers‐disease/symptoms‐causes/syc‐20350447

[7] Reynolds DS . A short perspective on the long road to effective treatments for Alzheimer's disease. Br J Pharmacol. 2019;176:3636‐3648. 30657599 10.1111/bph.14581 PMC6715596

[8] Haake A , Nguyen K , Friedman L , Chakkamparambil B , Grossberg GT . An update on the utility and safety of cholinesterase inhibitors for the treatment of Alzheimer's disease. Expert Opin Drug Saf. 2020;19:147‐157. 31976781 10.1080/14740338.2020.1721456

[9] Drummond E , Wisniewski T . Alzheimer's disease: experimental models and reality. Acta Neuropathol. 2017;133:155‐175. 28025715 10.1007/s00401-016-1662-x PMC5253109

[10] Warren WC , Jasinska AJ , García‐Pérez R , et al. The genome of the vervet (Chlorocebus aethiops sabaeus). Genome Res. 2015;25:1921‐1933. 26377836 10.1101/gr.192922.115 PMC4665013

[11] Palmour RM , Mulligan J , Howbert JJ , Ervin F . Of monkeys and men: vervets and the genetics of human‐like behaviors. Am J Hum Genet. 1997;61:481‐488. 9326311 10.1086/515526 PMC1715973

[12] Lemere CA , Beierschmitt A , Iglesias M , et al. Alzheimer's disease abeta vaccine reduces central nervous system abeta levels in a non‐human primate, the Caribbean vervet. Am J Pathol. 2004;165:283‐297. 15215183 10.1016/s0002-9440(10)63296-8 PMC1618542

[13] Farrer LA , Cupples LA , Haines JL , et al. Effects of age, sex, and ethnicity on the association between apolipoprotein E genotype and Alzheimer disease. A meta‐analysis. APOE and Alzheimer Disease Meta Analysis Consortium. JAMA. 1997;278:1349‐1356. 9343467

[14] Price DL , Martin LJ , Sisodia SS , et al. Aged non‐human primates: an animal model of age‐associated neurodegenerative disease. Brain Pathol. 1991;1:287‐296. 1688300 10.1111/j.1750-3639.1991.tb00672.x

[15] Fainman J , Eid M‐D , Ervin FR , Palmour RM . A primate model for Alzheimer's disease: investigation of the apolipoprotein E profile of the vervet monkey of St. Kitts. Am J Med Genet B Neuropsychiatr Genet. 2007;144B:818‐819. 17373728 10.1002/ajmg.b.30276

[16] Podlisny MB , Tolan DR , Selkoe DJ . Homology of the amyloid beta protein precursor in monkey and human supports a primate model for beta amyloidosis in Alzheimer's disease. Am J Pathol. 1991;138:1423‐1435. 1905108 PMC1886384

[17] Frye BM , Craft S , Latimer CS , et al. Aging‐related Alzheimer's disease‐like neuropathology and functional decline in captive vervet monkeys (Chlorocebus aethiops sabaeus). Am J Primatol. 2021;83:e23260. 33818801 10.1002/ajp.23260 PMC8626867

[18] Cramer PE , Gentzel RC , Tanis KQ , et al. Aging African green monkeys manifest transcriptional, pathological, and cognitive hallmarks of human Alzheimer's disease. Neurobiol Aging. 2018;64:92‐106. 29353102 10.1016/j.neurobiolaging.2017.12.011

[19] Jasinska AJ , Pandrea I , He T , et al. Immunosuppressive effect and global dysregulation of blood transcriptome in response to psychosocial stress in vervet monkeys (Chlorocebus sabaeus). Sci Rep. 2020;10:3459. 32103041 10.1038/s41598-020-59934-z PMC7044305

[20] Negrey JD , Dobbins DL , Howard TD , et al. Transcriptional profiles in olfactory pathway‐associated brain regions of African green monkeys: associations with age and Alzheimer's disease neuropathology. Alzheimers Dement. 2022;8:e12358. 10.1002/trc2.12358 PMC9609452 36313967

[21] Brown JN , Ortiz GM , Angel TE , et al. Morphine produces immunosuppressive effects in nonhuman primates at the proteomic and cellular levels. Mol Cell Proteomics. 2012;11:605‐618. 22580588 10.1074/mcp.M111.016121 PMC3434775

[22] Cox LA , Chan J , Rao P , et al. Integrated omics analysis reveals sirtuin signaling is central to hepatic response to a high fructose diet. Bmc Genomics [Electronic Resource]. 2021;22:870. 34861817 10.1186/s12864-021-08166-0 PMC8641221

[23] Taylor JR , Roth RH . Cognitive and motor deficits in the performance of an object retrieval task with a barrier‐detour in monkeys (Cercopithecus aethiops sabaeus) treated with MPTP: long‐term performance and effect of transparency of the barrier. Behav Neurosci. 1990;104:564‐576. 2206426 10.1037//0735-7044.104.4.564

[24] Diamond A , Zola‐Morgan S , Squire LR . Successful performance by monkeys with lesions of the hippocampal formation on AB and object retrieval, two tasks that mark developmental changes in human infants. Behav Neurosci. 1989;103:526‐537. 2736067 10.1037//0735-7044.103.3.526

[25] Wu T , Hu E , Xu S , et al. clusterProfiler 4.0: a universal enrichment tool for interpreting omics data. Innovation (Camb). 2021;2(3):100141. 34557778 10.1016/j.xinn.2021.100141 PMC8454663

[26] Yu G , Wang L‐G , Han Y , He Q‐Y . clusterProfiler: an R package for comparing biological themes among gene clusters. OMICS. 2012;16:284‐287. 22455463 10.1089/omi.2011.0118 PMC3339379

[27] Yu G , He Q‐Y . ReactomePA: an R/Bioconductor package for reactome pathway analysis and visualization. Mol Biosyst. 2016;12:477‐479. 26661513 10.1039/c5mb00663e

[28] Newman MEJ . Modularity and community structure in networks. Proc Natl Acad Sci USA. 2006;103:8577‐8582. 16723398 10.1073/pnas.0601602103 PMC1482622

[29] Loftus JC , Dhruv H , Tuncali S , et al. TROY (TNFRSF19) promotes glioblastoma survival signaling and therapeutic resistance. Mol Cancer Res. 2013;11:865‐874. 23699535 10.1158/1541-7786.MCR-13-0008 PMC3748253

[30] Tam SJ , Richmond DL , Kaminker JS , et al. Death receptors DR6 and TROY regulate brain vascular development. Dev Cell. 2012;22:403‐417. 22340501 10.1016/j.devcel.2011.11.018

[31] Ramos‐Martinez E , Ramos‐Martínez I , Molina‐Salinas G , Zepeda‐Ruiz WA , Cerbon M . The role of prolactin in central nervous system inflammation. Rev Neurosci. 2021;32:323‐340. 33661585 10.1515/revneuro-2020-0082

[32] Torner L . Actions of prolactin in the brain: from physiological adaptations to stress and neurogenesis to psychopathology. Front Endocrinol. 2016;7:25. 10.3389/fendo.2016.00025 PMC4811943 27065946

[33] Li X , Long J , He T , Belshaw R , Scott J . Integrated genomic approaches identify major pathways and upstream regulators in late onset Alzheimer's disease. Sci Rep. 2015;5:12393. 26202100 10.1038/srep12393 PMC4511863

[34] Lewczuk P , Matzen A , Blennow K , et al. Cerebrospinal fluid Aβ42/40 corresponds better than Aβ42 to amyloid PET in Alzheimer's disease. J Alzheimers Dis. 2017;55:813‐822. 27792012 10.3233/JAD-160722 PMC5147502

[35] Amft M , Ortner M , Eichenlaub U , et al. The cerebrospinal fluid biomarker ratio Aβ42/40 identifies amyloid positron emission tomography positivity better than Aβ42 alone in a heterogeneous memory clinic cohort. Alzheimers Res Ther. 2022;14:60. 35473631 10.1186/s13195-022-01003-w PMC9044878

[36] Shapiro IP , Masliah E , Saitoh T . Altered protein tyrosine phosphorylation in Alzheimer's disease. J Neurochem. 1991;56:1154‐1162. 1705956 10.1111/j.1471-4159.1991.tb11405.x

[37] Ciltas AC , Karabulut S , Sahin B , et al. FGF‐18 alleviates memory impairments and neuropathological changes in a rat model of Alzheimer's disease. Neuropeptides. 2023;101:102367. 37506425 10.1016/j.npep.2023.102367

[38] Shao Z , Browning JL , Lee X , et al. TAJ/TROY, an orphan TNF receptor family member, binds Nogo‐66 receptor 1 and regulates axonal regeneration. Neuron. 2005;45:353‐359. 15694322 10.1016/j.neuron.2004.12.050

[39] Kong M , Kim Y , Lee C . A strong synergistic epistasis between FAM134B and TNFRSF19 on the susceptibility to vascular dementia. Psychiatr Genet. 2011;21:37‐41. 21127458 10.1097/YPG.0b013e3283413496

[40] Stephan AH , Madison DV , Mateos JM , et al. A dramatic increase of C1q protein in the CNS during normal aging. J Neurosci. 2013;33:13460‐13474. 23946404 10.1523/JNEUROSCI.1333-13.2013 PMC3742932

[41] Baird GS , Nelson SK , Keeney TR , et al. Age‐dependent changes in the cerebrospinal fluid proteome by slow off‐rate modified aptamer array. Am J Pathol. 2012;180:446‐456. 22122984 10.1016/j.ajpath.2011.10.024 PMC3349859

[42] Wesenhagen KEJ , Gobom J , Bos I , et al. Effects of age, amyloid, sex, and ε4 on the CSF proteome in normal cognition. Alzheimers Dement. 2022;14:e12286. 10.1002/dad2.12286 PMC9074716 35571963

[43] Li J‐S , Yao Z‐X . Modulation of FGF receptor signaling as an intervention and potential therapy for myelin breakdown in Alzheimer's disease. Med Hypotheses. 2013;80:341‐344. 23321060 10.1016/j.mehy.2012.12.008

[44] Savas S , Tayfur E , Sarac F , et al. Human APRIL and FGF‐21 and adhesion molecules in relation to cognitive function in elderly diabetic patients. Int J Diabetes Dev Ctries. 2020;40:525‐5231.

[45] Drew DA , Tighiouart H , Scott TM , et al. FGF‐23 and cognitive performance in hemodialysis patients. Hemodial Int. 2014;18:78‐86. 24164913 10.1111/hdi.12100 PMC4443906

[46] Andres R , Forgie A , Wyatt S , Chen Q , de Sauvage FJ , Davies AM . Multiple effects of artemin on sympathetic neurone generation, survival and growth. Development. 2001;128:3685‐3695. 11585795 10.1242/dev.128.19.3685

[47] Honma Y , Araki T , Gianino S , et al. Artemin is a vascular‐derived neurotropic factor for developing sympathetic neurons. Neuron. 2002;35:267‐282. 12160745 10.1016/s0896-6273(02)00774-2

[48] Khosravi Z , Nasiri Khalili MA , Moradi S , Hassan Sajedi R , Zeinoddini M . The molecular chaperone artemin efficiently blocks fibrillization of TAU protein in vitro. Cell J. 2018;19:569‐577. 29105391 10.22074/cellj.2018.4510 PMC5672095

[49] Marvastizadeh N , Dabirmanesh B , Sajedi RH , Khajeh K . Anti‐amyloidogenic effect of artemin on α‐synuclein. Biol Chem. 2020;401:1143‐1151. 32673279 10.1515/hsz-2019-0446

[50] Chiavellini P , Canatelli‐Mallat M , Lehmann M , Goya RG , Morel GR . Therapeutic potential of glial cell line‐derived neurotrophic factor and cell reprogramming for hippocampal‐related neurological disorders. Neural Regen Res. 2022;17:469‐476. 34380873 10.4103/1673-5374.320966 PMC8504380

[51] Straten G , Eschweiler GW , Maetzler W , Laske C , Leyhe T . Glial cell‐line derived neurotrophic factor (GDNF) concentrations in cerebrospinal fluid and serum of patients with early Alzheimer's disease and normal controls. J Alzheimers Dis. 2009;18:331‐337. 19584438 10.3233/JAD-2009-1146

[52] Muenchhoff J , Poljak A , Song F , et al. Plasma protein profiling of mild cognitive impairment and Alzheimer's disease across two independent cohorts. J Alzheimers Dis. 2015;43:1355‐1373. 25159666 10.3233/JAD-141266

[53] Miyakis S , Giannakopoulos B , Krilis SA . Beta 2 glycoprotein I–function in health and disease. Thromb Res. 2004;114:335‐346. 15507263 10.1016/j.thromres.2004.07.017

[54] Mather KA , Thalamuthu A , Oldmeadow C , et al. Genome‐wide significant results identified for plasma apolipoprotein H levels in middle‐aged and older adults. Sci Rep. 2016;6:23675. 27030319 10.1038/srep23675 PMC4814826

[55] Song F , Poljak A , Crawford J , et al. Plasma apolipoprotein levels are associated with cognitive status and decline in a community cohort of older individuals. PLoS One. 2012;7:e34078. 22701550 10.1371/journal.pone.0034078 PMC3372509

[56] Xu C , Garcia D , Lu Y , et al. Levels of angiotensin‐converting enzyme and apolipoproteins are associated with Alzheimer's disease and cardiovascular diseases. Cells. 2021;11. doi:10.3390/cells11010029 PMC8744784 35011591

[57] Arvanitakis Z , Brey RL , Rand JH , et al. Relation of antiphospholipid antibodies to postmortem brain infarcts in older people. Circulation. 2015;131:182‐189. 25301832 10.1161/CIRCULATIONAHA.114.012479 PMC4293251

[58] Muenchhoff J , Song F , Poljak A , et al. Plasma apolipoproteins and physical and cognitive health in very old individuals. Neurobiol Aging. 2017;55:49‐60. 28419892 10.1016/j.neurobiolaging.2017.02.017

[59] Wennberg AMV , Hagen CE , Petersen RC , Mielke MM . Trajectories of plasma IGF‐1, IGFBP‐3, and their ratio in the Mayo Clinic Study of Aging. Exp Gerontol. 2018;106:67‐73. 29474865 10.1016/j.exger.2018.02.015 PMC5911407

[60] van Varsseveld NC , Sohl E , Drent ML , Lips P . Gender‐specific associations of serum insulin‐like growth factor‐1 with bone health and fractures in older persons. J Clin Endocrinol Metab. 2015;100:4272‐4281. 26323023 10.1210/jc.2015-2549

[61] Holzenberger M , Dupont J , Ducos B , et al. IGF‐1 receptor regulates lifespan and resistance to oxidative stress in mice. Nature. 2003;421:182‐187. 12483226 10.1038/nature01298

[62] Carro E , Trejo JL , Gomez‐Isla T , LeRoith D , Torres‐Aleman I . Serum insulin‐like growth factor I regulates brain amyloid‐beta levels. Nat Med. 2002;8:1390‐1397. 12415260 10.1038/nm1202-793

[63] Arend WP . The balance between IL‐1 and IL‐1Ra in disease. Cytokine Growth Factor Rev. 2002;13:323‐340. 12220547 10.1016/s1359-6101(02)00020-5

[64] Parker H , Ellison SM , Holley RJ , et al. Haematopoietic stem cell gene therapy with IL‐1Ra rescues cognitive loss in mucopolysaccharidosis IIIA. EMBO Mol Med. 2020;12:e11185. 32057196 10.15252/emmm.201911185 PMC7059006

[65] Lotrich FE , Butters MA , Aizenstein H , Marron MM , Reynolds CF 3rd , Gildengers AG . The relationship between interleukin‐1 receptor antagonist and cognitive function in older adults with bipolar disorder. Int J Geriatr Psychiatry. 2014;29:635‐644. 24273017 10.1002/gps.4048 PMC4013203

[66] Hope S , Hoseth E , Dieset I , et al. Inflammatory markers are associated with general cognitive abilities in schizophrenia and bipolar disorder patients and healthy controls. Schizophr Res. 2015;165:188‐194. 25956633 10.1016/j.schres.2015.04.004

[67] Blacker D , Wilcox MA , Laird NM , et al. Alpha‐2 macroglobulin is genetically associated with Alzheimer disease. Nat Genet. 1998;19:357‐360. 9697696 10.1038/1243

[68] Kovacs DM . alpha2‐macroglobulin in late‐onset Alzheimer's disease. Exp Gerontol. 2000;35:473‐479. 10959035 10.1016/s0531-5565(00)00113-3

[69] Varma VR , Varma S , An Y , et al. Alpha‐2 macroglobulin in Alzheimer's disease: a marker of neuronal injury through the RCAN1 pathway. Mol Psychiatry. 2017;22:13‐23. 27872486 10.1038/mp.2016.206 PMC5726508

[70] Zijlstra M , Bix M , Simister NE , Loring JM , Raulet DH , Jaenisch R . Beta 2‐microglobulin deficient mice lack CD4‐8+ cytolytic T cells. Nature. 1990;344:742‐746. 2139497 10.1038/344742a0

[71] Smith LK , He Y , Park J‐S , et al. β2‐microglobulin is a systemic pro‐aging factor that impairs cognitive function and neurogenesis. Nat Med. 2015;21:932‐937. 26147761 10.1038/nm.3898 PMC4529371

[72] Qun S , Hu F , Wang G , et al. Serum beta2‐microglobulin levels are highly associated with the risk of acute ischemic stroke. Sci Rep. 2019;9:6883. 31053801 10.1038/s41598-019-43370-9 PMC6499788

[73] Doecke JD , Laws SM , Faux NG , et al. Blood‐based protein biomarkers for diagnosis of Alzheimer disease. Arch Neurol. 2012;69:1318‐1325. 22801742 10.1001/archneurol.2012.1282 PMC6287606

[74] Dominici R , Finazzi D , Polito L , et al. Comparison of β2‐microglobulin serum level between Alzheimer's patients, cognitive healthy and mild cognitive impaired individuals. Biomarkers. 2018;23:603‐608. 29741401 10.1080/1354750X.2018.1468825

[75] Carrette O , Demalte I , Scherl A , et al. A panel of cerebrospinal fluid potential biomarkers for the diagnosis of Alzheimer's disease. Proteomics. 2003;3:1486‐1494. 12923774 10.1002/pmic.200300470

[76] Zhao Y , Zheng Q , Hong Y , et al. β‐Microglobulin coaggregates with Aβ and contributes to amyloid pathology and cognitive deficits in Alzheimer's disease model mice. Nat Neurosci. 2023;26:1170‐1184. 37264159 10.1038/s41593-023-01352-1

[77] Tan J , Town T , Mullan M . CD40‐CD40L interaction in Alzheimer's disease. Curr Opin Pharmacol. 2002;2:445‐451. 12127879 10.1016/s1471-4892(02)00180-7

[78] Ots HD , Tracz JA , Vinokuroff KE , Musto AE . CD40–CD40L in neurological disease. Int J Mol Sci. 2022;23(8):4115. doi:10.3390/ijms23084115 35456932 PMC9031401

[79] Yalcin AD , Gorczynski RM , Kahraman MS , Demirel MU , Terzioglu E . CD40, CD45 CTLA‐4 levels are elevated in healthy older adults. Clin Lab. 2012;58:449‐456. 22783574

[80] Ait‐ghezala G , Abdullah L , Volmar C‐H , et al. Diagnostic utility of APOE, soluble CD40, CD40L, and Abeta1‐40 levels in plasma in Alzheimer's disease. Cytokine. 2008;44:283‐287. 18835787 10.1016/j.cyto.2008.08.013

[81] Cox PA , Davis DA , Mash DC , Metcalf JS , Banack SA . Dietary exposure to an environmental toxin triggers neurofibrillary tangles and amyloid deposits in the brain. Proc R Soc B Biol Sci. 2016;283(1823):20152397. doi:10.1098/rspb.2015.2397 PMC4795023 26791617

[82] Latimer CS , Shively CA , Keene CD , et al. A nonhuman primate model of early alzheimer's disease pathologic change: implications for disease pathogenesis. Alzheimer Dement. 2018;15(1):93‐105. doi:10.1016/j.jalz.2018.06.3057 PMC6383152 30467082

