# Supplementary material for: Plasma and CSF biomarkers of aging and cognitive decline in Caribbean vervets
Source: Alzheimers Dement. 2024 Jul 1;20(8):5460–80. doi: 10.1002/alz.14038 (PMC11350037; doi:10.1002/alz.14038)
Supplement: Supplementary file 1 — Supporting Information [file ALZ-20-5460-s004.pdf]

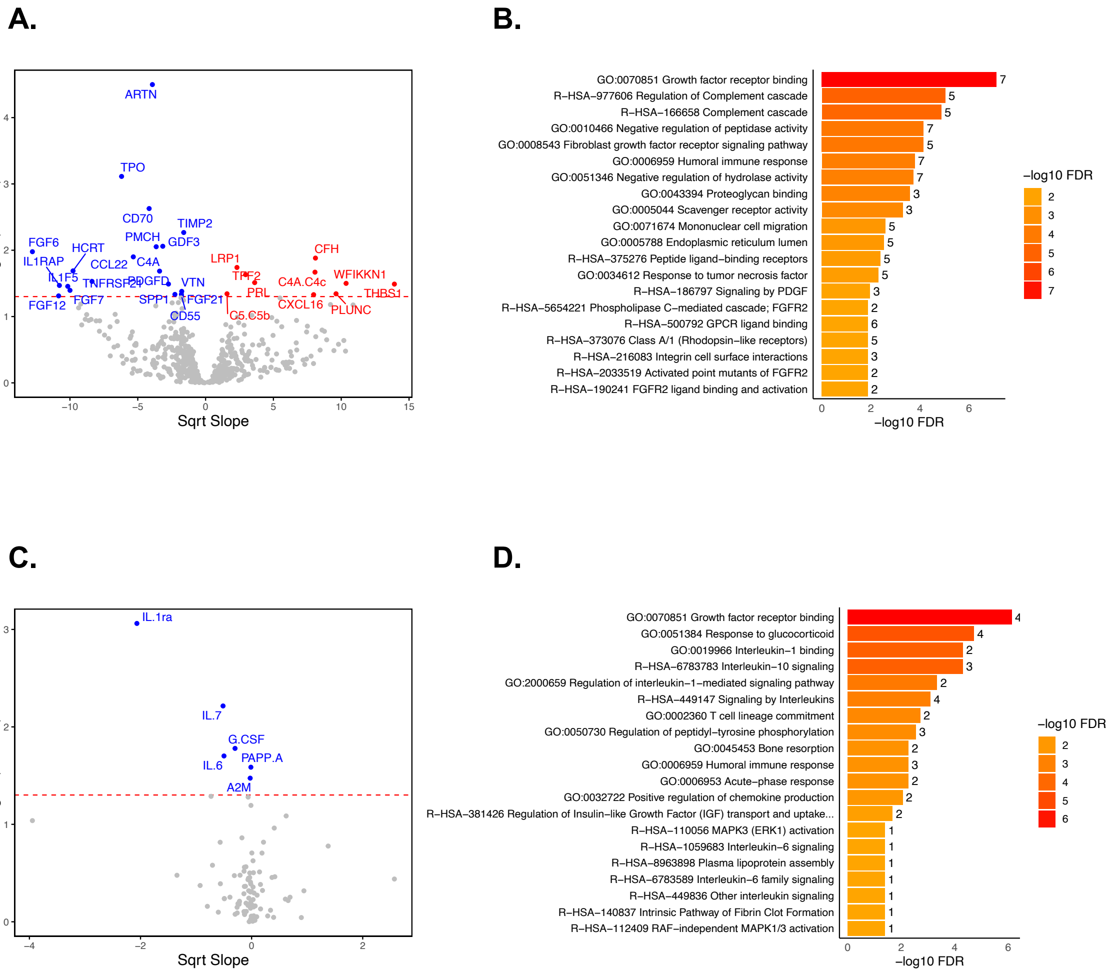

Supplemental Fig. 1: Additional analyses of CSF and plasma proteins that associated with overall performance on ORT (percentage of success on sub-components relative to total number of tests). **A,** Volcano plot of CSF proteins that significantly changed with respect to overall performance on ORT sub-components. **B,** Bar plot of top pathways of significant CSF proteins. **C,** Volcano plot of plasma proteins that significantly changed with overall performance on ORT sub-components. **D,** Bar plot of top pathways of significant plasma proteins.
