# Supplementary material for: Plasma and CSF biomarkers of aging and cognitive decline in Caribbean vervets
Source: Alzheimers Dement. 2024 Jul 1;20(8):5460–80. doi: 10.1002/alz.14038 (PMC11350037; doi:10.1002/alz.14038)
Supplement: Supplementary file 2 — Supporting Information [file ALZ-20-5460-s003.pdf]

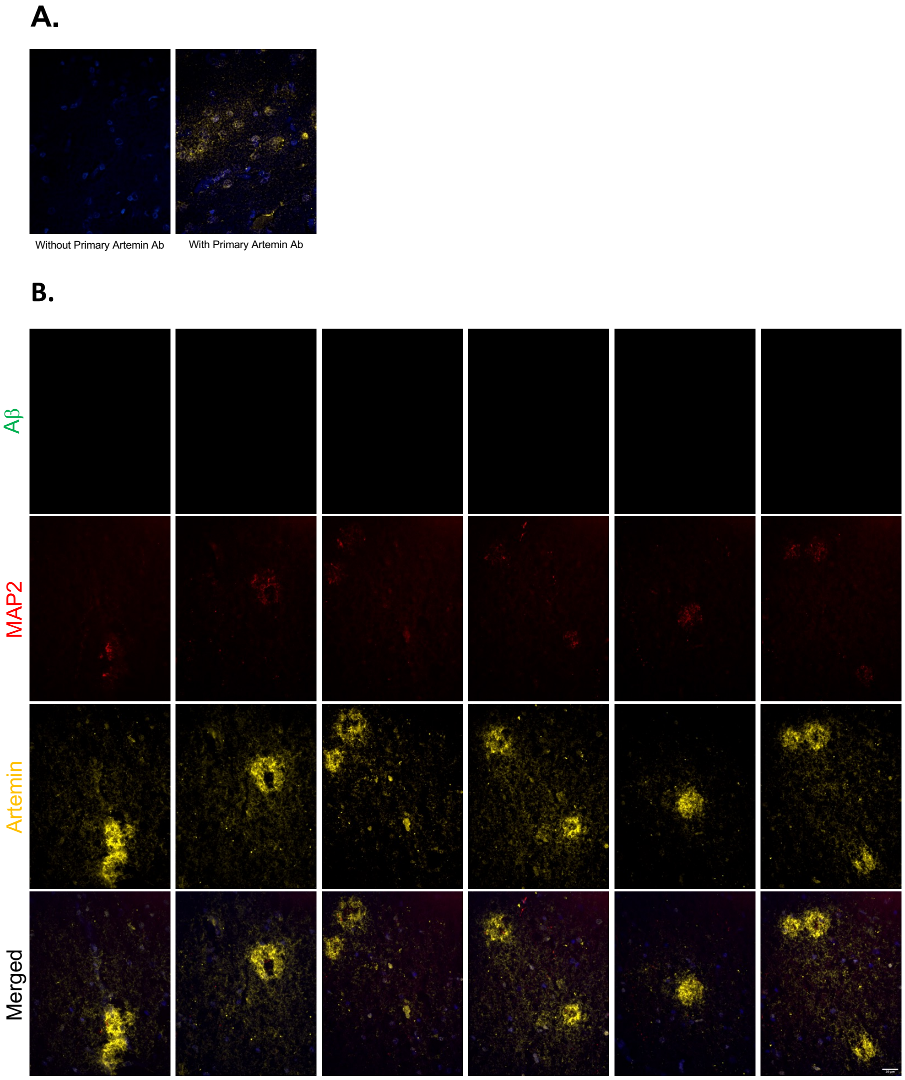

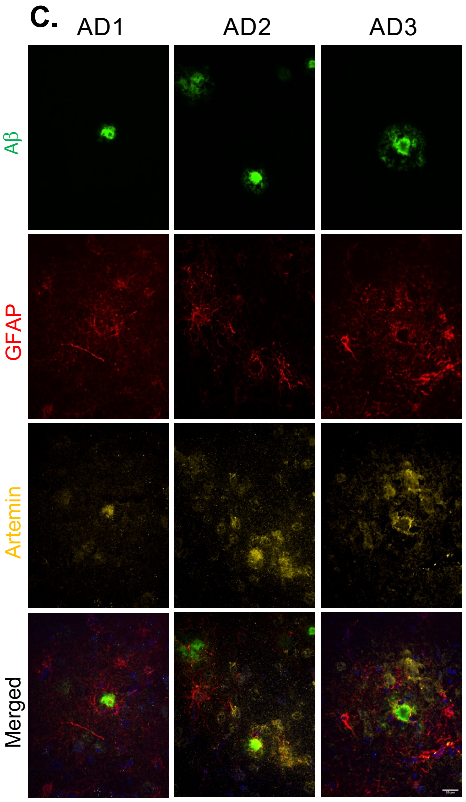

Supplemental Fig. 2: Additional Artemin Staining Patterns in Human Brains. **A,** Omission of primary antibody staining on the left and Artemin staining on the right confirmed specificity of the staining. **B,** Artemin staining colocalized with MAP2 staining even in the absence of Aβ IR in an aged control brain. **C,** Artemin staining was not clearly colocalized with GFAP in Human AD brains.
